# Oxidized Lipids in Persistent Pain States

**DOI:** 10.3389/fphar.2019.01147

**Published:** 2019-10-15

**Authors:** Tabea Osthues, Marco Sisignano

**Affiliations:** ^1^Fraunhofer Institute for Molecular Biology and Applied Ecology IME, Branch for Translational Medicine and Pharmacology TMP, Frankfurt, Germany; ^2^Institute of Clinical Pharmacology, Pharmazentrum Frankfurt/ZAFES, University Hospital, Goethe-University, Frankfurt, Germany

**Keywords:** pain, transient receptor potential channels, linoleic acid metabolites, eicosanoids, HODE, lipids inflammatory pain, neuropathic pain

## Abstract

Chemotherapy, nerve injuries, or diseases like multiple sclerosis can cause pathophysiological processes of persistent and neuropathic pain. Thereby, the activation threshold of ion channels is reduced in peripheral sensory neurons to normally noxious stimuli like heat, cold, acid, or mechanical due to sensitization processes. This leads to enhanced neuronal activity, which can result in mechanical allodynia, cold allodynia, thermal hyperalgesia, spontaneous pain, and may initiate persistent and neuropathic pain. The treatment options for persistent and neuropathic pain patients are limited; for about 50% of them, current medication is not efficient due to severe side effects or low response to the treatment. Therefore, it is of special interest to find additional treatment strategies. One approach is the control of neuronal sensitization processes. Herein, signaling lipids are crucial mediators and play an important role during the onset and maintenance of pain. As preclinical studies demonstrate, lipids may act as endogenous ligands or may sensitize transient receptor potential (TRP)-channels. Likewise, they can cause enhanced activity of sensory neurons by mechanisms involving G-protein coupled receptors and activation of intracellular protein kinases. In this regard, oxidized metabolites of the essential fatty acid linoleic acid, 9- and 13-hydroxyoctadecadienoic acid (HODE), their dihydroxy-metabolites (DiHOMEs), as well as epoxides of linoleic acid (EpOMEs) and of arachidonic acid (EETs), as well as lysophospholipids, sphingolipids, and specialized pro-resolving mediators (SPMs) have been reported to play distinct roles in pain transmission or inhibition. Here, we discuss the underlying molecular mechanisms of the oxidized linoleic acid metabolites and eicosanoids. Furthermore, we critically evaluate their role as potential targets for the development of novel analgesics and for the treatment of persistent or neuropathic pain.

## Introduction

Pain is considered to protect the organism of tissue damage and harm (Kuner and Flor, 2016; Peirs and Seal, 2016). Such harm and potentially damaging stimuli can be thermal, chemical, or mechanical stimuli like heat, extreme cold, pressure, and chemicals (Dubin and Patapoutian, 2010). The stimuli are detected by peripheral sensory neurons of the somatosensory system, which arise in the skin, muscles, joints, and fascia (Colloca et al., 2017). Through activation of specific ion channels located in the plasma membrane of sensory neurons, like the transient receptor potential (TRP) and purinergic channels, the stimuli are converted into electrical activity (Kuner, 2010; Talbot et al., 2016). The transmission of the signal happens only in an all-or-none action potential manner and leads to pain perception, depending on the frequency and intensity (Dubin and Patapoutian, 2010). Thereby, the action potentials are transmitted *via* the peripheral axonal branch to the cell bodies of the nociceptors, located in the dorsal root ganglia (DRG), and then *via* the central axonal branch to the spinal cord (Basbaum et al., 2009). Here, after synaptic processing, the signal is transmitted into the central nervous system through the thalamus to the somatosensory cortex and associated areas (Scholz and Woolf, 2002).

### Pathophysiologic Pain States

Apart from its protective function, pain can become chronic when maladaptive processes such as persisting tissue damage or pathophysiological conditions after healed injury, provoke an unresolved sensitization (Basbaum et al., 2009; Kuner and Flor, 2016; Peirs and Seal, 2016). Pathophysiological conditions are highly diverse ranging from viral infection, inflammation, tumor growth, autoimmune diseases, metabolic disorders, and vascular diseases (Kuner and Flor, 2016) and involve signaling mediators, such as cytokines, chemokines, and lipids. The pain manifests with different qualities including stabbing, pricking, burning, or arching (Peirs and Seal, 2016). In this pathophysiological state, the activation threshold of sensory neurons is reduced, resulting in peripheral sensitization. Moreover, increased responsiveness of the postsynaptic neurons in the spinal cord may occur in pathophysiological states by increased synaptic activity and disinhibition of spinal nociceptive input. These plasticity changes in the spinal cord are defined as central sensitization, leading to increased pain perception (Latremoliere et al., 2015). Typically chronic pain states are characterized by hyperalgesia, which is defined as an increased response to painful thermal and mechanical stimuli as well as allodynia, where nociceptive responses occur to normally innocuous stimuli such as light touch (Ji et al., 2014; Lim and Kim, 2016). A special form of chronic pain is neuropathic pain which is characterized by neuronal damage of the peripheral or central nervous system through nerve injury, amputation, trauma, infections, toxic substances, or metabolic disorders, such as diabetes (Kobayashi et al., 2015; Kuner and Flor, 2016). However, neuropathic pain is not only provoked by nerve injury or tissue damage. It is also associated with an imbalance of activity in inflammatory pathways as response of the somatosensory, immune, autonomic, and vascular/circulatory system to tissue damage, pathogens, or irritants (Ji et al., 2016; Kuner and Flor, 2016). Additionally, neuropathic pain is characterized by mechanical allodynia and hyperalgesia, but can also involve spontaneous pain (Kobayashi et al., 2015). According to epidemiological estimations, 1 in 26 people worldwide suffers from neuropathic pain (van Hecke et al., 2014; Peirs and Seal, 2016).

### Current Treatment of Neuropathic Pain

Neuropathic pain is among the most difficult types of chronic pain to treat, which not only significantly impairs patients’ quality of life but also adds to the burden of direct and indirect medical cost for our society (Leung and Cahill, 2010; Colloca et al., 2017). Currently, there is a broad variety of treatment options for patients. They range from tricyclic antidepressants, serotonin-noradrenaline reuptake inhibitors, antiepileptics to botulinum toxin A, capsaicin, and opioids (Finnerup et al., 2015; Colloca et al., 2017). However, it is reported that the efficacy of those therapeutics is low, and the number needed to treat (NNT) for first-line treatments is considerably high (Finnerup et al., 2015). For example one in seven to eight patients treated with pregabalin, a calcium channel blocker, showed a significant effect in pain relief (Finnerup et al., 2015). Likewise, various studies demonstrate that less than 50% of patients experience satisfactory pain relief and suffer from accompanying side effects of neuropathic pain therapy (O’Connor and Dworkin, 2009; Tesfaye et al., 2013).

### Lipids as Alternative Treatment Option

Those numbers indicate a need for novel treatment options of persistent and neuropathic pain. During the past decades research has proceeded in identifying key mechanisms in signaling pathways and other alterations underlying neuropathic and chronic pain. For example, it was found that after peripheral nerve damage an extensive immune response occurs around the cell bodies of injured and uninjured sensory neurons (Calvo et al., 2012). This is accompanied not only by activation of resident immune cells and migration of immune cells, but also with increased concentrations of cytokines in the injured nervous tissue (Kiguchi et al., 2012). In this regard, signaling lipids of the arachidonic acid (AA) and linoleic acid (LA) pathway seem to be particular important mediators that are released by immune cells or neurons and act as paracrine mediators, by activation of G-protein coupled receptors (GPCRs) and/or modulating the activity of ion channels in peripheral sensory neurons (Sisignano et al., 2014). These activations mediate second messenger signaling cascades that lower the threshold of ion channels, including the transient receptor potential cation channel subfamily vanilloid 1 (TRPV1) channel, leading to increased activity of peripheral sensory neurons (Moran et al., 2011; Lim and Kim, 2016; Pinho-Ribeiro et al., 2017). Here, the TRPV1 channel is of special interest, because it plays not only a well-established role in inflammatory pain, but is also upregulated in different models of peripheral neuropathic pain both at dorsal root ganglion (DRG) peripheral and central synapses (Rivat et al., 2018).

It was shown before, that antagonizing TRPV1 leads to hyperthermia in humans (Gavva et al., 2008), but not in mice (Moran et al., 2011), which implies, that targeting TRPV1 is not a favorable strategy in the treatment of chronic or neuropathic pain.

Moreover, it is not desirable to silence or antagonize TRPV1 completely, because of its protective function in responding to noxious heat stimuli (Moran et al., 2011; Calvo et al., 2012; De Petrocellis et al., 2012). Therefore, an alternative strategy to target this channel may be to reduce its increased activity during chronic pain and to abolish its sensitization. In this regard, lipid mediators, their synthesis, metabolism, and signaling pathways may represent alternative targets to reduce excessive neuronal activity in chronic pain states. Endogenous lipids, including some oxidized lipid metabolites from linoleic acid and arachidonic acid, have previously been shown to cause nociceptor excitation and pain (Patwardhan et al., 2010; De Petrocellis et al., 2012; Gregus et al., 2012). Such biologically active metabolites are 9- and 13-hydroxyoctadecadienoic acid (HODE), both of which contribute to the heat responsiveness and sensitization of TRPV1, as earlier studies showed (Patwardhan et al., 2010).

In the last years further metabolites of the linoleic acid were investigated, like their dihydroxy-metabolites (DiHOMEs) and the epoxides of linoleic acid, the epoxy-octadecenoic acids (EpOMEs) regarding their role in nociception (Sisignano et al., 2012; Zimmer et al., 2018).

In this review we first discuss the effects of the different lipid metabolites in states of inflammation and pain. In the second part, we critically discuss their synthesizing and metabolizing enzymes or receptors as potential novel targets for the treatment of chronic and neuropathic pain in patients.

## Metabolism of Oxidized Lipids

The omega-6 fatty acids linoleic acid (LA) and arachidonic acid (AA) metabolites are of special interest as pain modulators, because abnormalities of lipid metabolism play a central role in various diseases, like type 1 diabetes, epilepsy, and inflammation as well as during pain (Coste TC et al., 2003; Inceoglu et al., 2008; Hung et al., 2015; Hohmann et al., 2017; Zimmer et al., 2018). In the following section, we focus on the effects of LA- and AA-derived lipids and their role in pain pathology.

**Figure 1:**
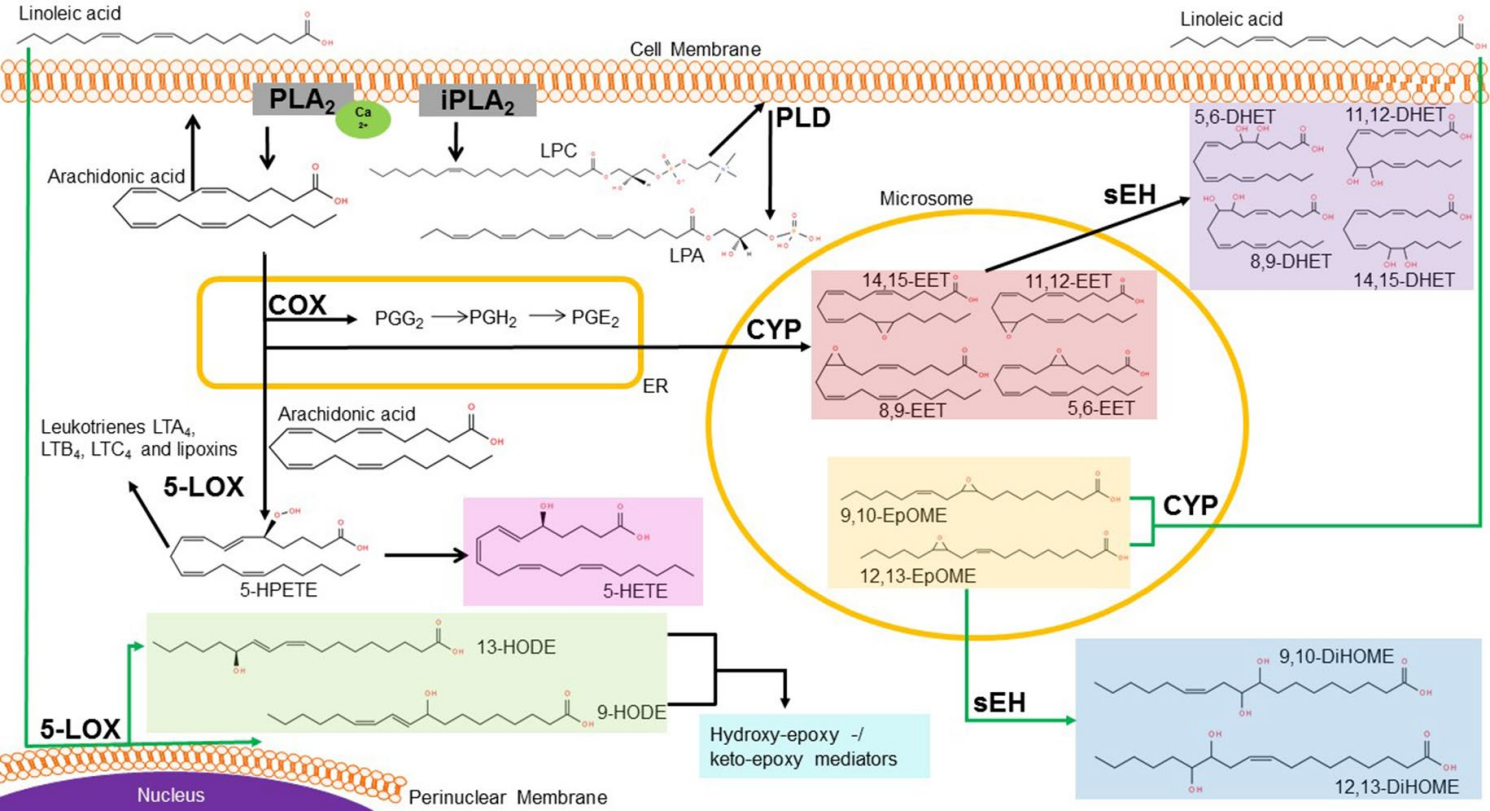
Metabolic pathway of linoleic and arachidonic acid. In the cell, linoleic acid (LA) is rapidly metabolized *via* different enzymes into various bioactive metabolites. Lipoxygenases (LOX) can generate 13- and 9-HODE at the perinuclear membrane of the nucleus, whereas the cytochrome-P_450_- enzymes (CYP) metabolize LA into EpOMEs. These are further metabolized to DiHOMEs *via* soluble epoxide hydrolase (sEH). In the endoplasmatic reticulum (ER), AA is the precursor of the cyclooxygenase (COX) products prostaglandins (e.g. PGE_2_). AA can also be metabolized to the epoxyeicosatrienoic acids (EETs), which are further metabolized by sEH to DHETs in the cytosol. At the plasma membrane, phospholipase A liberates either AA out of the phospholipids or generates lysophophatidylcholine (LPC). LPC is then metabolized through the extracellular phospholipase D (PLD) autotaxin to lysophosphatidic acid (LPA). Abbreviations: LTA, leukotriene A; LTB, leukotriene B; LTC, leukotriene C; LOX, lipoxygenase; 5-HPETE, arachidonic acid 5-hydroperoxide; 5-HETE, 5-Hydroxyeicosatetraenoic acid; 13-/9-HODE, 13-/9-Hydroxyoctadecadienoic acid; EET, epoxyeicosatrienoic acid; PG, prostaglandin; COX, cyclooxygenase; CYP, cytochrome enzymes; DHET, Dihydroxy eicosatrienoic acid; EpOME, Epoxy octadecenoic acid; DiHOME, Dihydroxy-octadecenoic acid; LPC, lysophosphatidylcholine, LPA, lysophosphatidic acid; PLD, phospholipase D; sEH, soluble epoxide hydrolase; ER, endoplasmatic reticulum. Source structural formula: http://lipidmaps.org/.

Linoleic acid is one of the essential ω-6 fatty acids, which leads to formation of the smooth, non-dry outer epidermal barrier upon dietary intake (Hansen et al., 1958; Gill and Valivety, 1997; Nakamura and Nara, 2003; Ramsden et al., 2017). After transport into the cell, delta-6 desaturase metabolize LA to γ-linoleic acid, being the substrate of elongase enzymes followed by delta-5 desaturase to form arachidonic acid in the endoplasmatic reticulum (Gill and Valivety, 1997; Nakamura and Nara, 2003; Russo, 2009). AA is ubiquitously present in human tissue, especially within phosphoplipids of the cell membrane. Liberation of AA is hydrolyzed upon stimulation of various cellular signaling pathways, for example in the context of inflammation through activation of the Ca^2+^-activated phospholipase A_2_ (cPLA_2_) (Davies et al., 1984; Murakami and Kudo, 2002; Spector and Kim, 2015) (**Figure 1**).

There are three major enzyme classes for further metabolization of AA. First, there are the cyclooxygenases (COX), which catalyze a bis-dioxygenation reaction to form the unstable endoperoxide intermediate prostaglandin G_2_ (PGG_2_) (Davies et al., 1984; Brenneis et al., 2011). PGG_2_ is then reduced by a hydroperoxidase to prostaglandin H_2_ and then either by various isomerases or non-enzymatic reactions into several prostanoids (Davies et al., 1984; Hiesinger et al., 2019). They act as bioactive mediators, for example prostaglandin E_2_ causes inflammatory pain by binding to its respective receptors in neurons, the EP receptor family, and subsequent activation of protein kinases and sensitization of TRPV1 (Kawabata, 2011).

The second class of metabolizing enzymes of AA are the lipoxygenases (LOX), in particular the 5-, 12- and 15-LOX. They produce hydroperoxyeicosatetraenoic acids (HPETEs) which are reduced by the glutathione peroxidase system to the corresponding monohydroxyeicosatetraenoic acids (HETEs) (Davies et al., 1984; Hiesinger et al., 2019). Thus, 5-HPETE is further metabolized by 5-LOX into leukotriene A_4_, being the precursor of leukotriene B_4_ and C_4_ and lipoxins (Davies et al., 1984; Russo, 2009; Hiesinger et al., 2019). Leukotrienes are well known for their role in asthma and allergy, whereas lipoxins play a role in the resolution of inflammation and can also be produced by 12- and 15-LOX (Dennis and Norris, 2015; Hiesinger et al., 2019) (**Figure 1**).

Additionally, LOX enzymes can oxygenate LA by oxidizing the carbons at position 9 or 13 of its backbone and by this produce 9S- and 13S-hydroperoxides, like 9- or 13- hydroxyoctadecadienoic acid (HODE) (Oliw, 1994; Andreou et al., 2009; Newcomer and Brash, 2015). 9- and 13-HODE are released in skin upon temperature increase leading to heat responsiveness of TRPV1 *in vitro* and *in vivo* (Patwardhan et al., 2010). Through hydroxylation of the ω-side chain of LA 18-, 17- and 16-HODE are formed, while allylic hydroxylation yields in 8-, 11- and 14-HODE (Oliw, 1994).

The cytochrome P450 (CYP450) enzymes are the third class of AA-metabolizing enzymes, which are primarily monooxygenases with some substrate selectivity (Hiesinger et al., 2019). There are two forms of CYP enzymes, the hydroxylases and the epoxygenases. Latter ones, namely isoforms of the families CYP2C and CYP2J, convert the AA to epoxyeicosatrienoic acids (EETs) (Spector et al., 2004; Fleming, 2007; Hiesinger et al., 2019). In this reaction, one of the four double bonds of AA is oxidized to an epoxide. Depending on the site of oxygenation, this leads to the production of one of the four regioisomers: 5,6-EET, 8,9-EET, 11,12-EET or 14,15-EET (Marowsky et al., 2009; Sisignano et al., 2012; Mule et al., 2017). EETs can be released from cells to act as paracrine signaling mediators (Sisignano et al., 2012). In various studies, EETs were shown to decrease inflammatory pain, but they may also have pronociceptive effects at certain concentrations and time points during onset or maintenance of persistent pain (Inceoglu et al., 2006) (see **Figure 1**).

Both, LOX enzymes and CYP epoxygenases, can metabolize LA as further substrate, to generate epoxyoctadecenoic acids (EpOMEs) (Zhang et al., 2014). The EpOMEs and the EETs are rapidly converted by the soluble epoxide hydrolase (sEH) to pro-inflammatory dihydroxyoctadecenoic acids (DiHOMEs) and dihydroeicosatrienoic acids (DHETs) (Inceoglu et al., 2006). The latter one are reported to have lower activities than their corresponding EETs (Wagner et al., 2011). In contrast, a more toxic effect was reported for the DiHOMEs than for the EpOMEs (Moghaddam et al., 1997). The contradictory effects of oxidized LA- and AA metabolites in persistent pain require a more detailed analysis to identify the individual role of each lipid mediator in pain pathology. Furthermore, a detailed analysis helps to identify targets within lipid synthesis, metabolism and signaling that may give rise to novel analgesics. In the last decades, accumulating evidence suggests a central role of LA and AA metabolites in the regulation of persistent and neuropathic pain as well as involved pathways. In the following passage we critically discuss the effects of these lipids with focus on epoxy- and dihydroxy-metabolites of arachidonic acid (EETs and DHETs) and linoleic acid (EpOMEs and DiHOMEs).

## The Roles of Oxidized Lipids in Inflammation and Pain

### EETs

#### 5,6-EET

Neuroinflammation is a characteristic feature of neuropathic and persistent pain (Ji et al., 2016). Thus, metabolites of AA with effects on inflammatory and neuropathic pain are of special interest in pain research. One of them, the 5,6-EET regioisomer, has dilatatory effects on cerebral arterioles *in vivo* indicating a proinflammatory role since increased blood flow to the inflamed site and extravasation of fluid, plasma protein, and leukocytes are features of inflammation (Pober and Sessa, 2014; Dennis and Norris, 2015). Proinflammatory cytokines such as interleukin (IL)-6 and tumor necrosis factor (TNF)-α were found to be elevated in macrophages after 5,6-EET and lipopolysaccharide (LPS) treatment, confirming the proinflammatory effect of 5,6-EET (Zhang et al., 2018).

Unfortunately, very little is known about the functions of 5,6-EET in neuroinflammation and neuropathic pain, because it is an unstable metabolite of AA and undergoes rapid hydrolysis (Zhang et al., 2014). Interestingly, cupping therapy, which is used against muscle pain and inflammation, resulted in elevated levels of 5,6-EET in skin of treated mice, whereas the concentrations of its corresponding diol 5,6-DHET were not increased (Zhang et al., 2018).

After application of all four EETs in LPS-induced inflammation a pronociceptive effect was observed (Inceoglu et al., 2006). On the other hand, the EETs reversed thermal hyperalgesia, which could be due to the mixture of all four EET regioisomers (Inceoglu et al., 2006). Moreover, it was reported that 5,6-EET is synthesized in dorsal root ganglia (DRG) and spinal cord during nociceptive activity after induction of acute pain. This resulted in activation of the ligand-gated ion channel transient receptor potential ankyrin 1 (TRPA1) and subsequent release of the proinflammatory neuropeptide calcitonin gene-related peptide (CGRP) from peripheral nerves causing mechanical allodynia (Sisignano et al., 2012). These results imply a role for 5,6-EET in the transition between acute and persistent pain (**Table 1A**). However, a direct binding of 5,6-EET to TRPA1 was not shown.

Apart from TRPA1, 5,6- EET seems to activate another member of the family of TRP channels. It was reported that the temperature sensor TRPV4 acts as a receptor for 5,6-EET and gets activated through binding of 5,6-EET (Watanabe et al., 2003) (**Table 1A**).

**Table 1A:** Metabolites of arachidonic acid (AA) in inflammation and pain.

Animals/tissue/cell type	Effect	Signaling	Experiment	Experimental details	Target	Refs.
5,6-EET						
Cerebral arterioles of tsA-201 cells, Ca_V_3.1 KO and Ca_V_3.2 KO mice, tsA-201 cells	Dilation	Inhibition of T-type calcium channels	*In vivo, in vitro*	Transfection, electrophysiological recordings, microperfusion of mesenteric resistance arteries	Ca_v_3.1, Ca_v_3.2, Ca_v_3.3	(Dennis et al., 2011; Cazade et al., 2014)
Nude and C57Bl/6 mice, RAW264.7 cells, bone marrow derived macrophages	Proinflammatory	Proinflammatory cytokine production	*In vitro, in vivo cupping therapy*	Ultra-performance liquid chromatography – tandem mass spectrometry (UPLC-MS/MS), cupping treatment, enzyme-linked immunosorbent assay (ELISA), fluorescence-activated cell sorting (FACS) analysis		(Zhang et al., 2018)
bovine aortic endothelial cells, HUVECs, 3T3-L1		Activation of PPARγ-regulated transcription	*In vitro*	Transfection, PPARγ-competition binding assay with [^3^H]rosiglitazone, plate flow for laminar shear stress experiments, quantitative real-time polymerase chain reaction (qPCR), sEH activity assay, reverse-phase high performance liquid chromatography (HPLC), lipid quantification by Quattro Ultima tandem quadrupole mass spectrometer, immunoblotting analysis	PPARγ	(Liu et al., 2005; Hiesinger et al., 2019)
pulmonary murine microvascular endothelial cells	no	PI3K/Akt pathway activation, proliferation, migration	*In vitro*	stimulation with EETs/DHETs and quantification with NICI/GC/MS, proliferation assay with [^3^H]thymidine, transwell migration assay, matrigel-based capillary formation assay, Western Blot, qPCR, *in vivo* angiogenesis in subcutaneous sponge model, immunofluorescence of tumors from human non-small cell lung cancer cells		(Pozzi et al., 2005)
CHO-K1 cells, BV-2 microglial cells, BALB/c mice brain tissue		Activation of CB_2_ receptor	*In vitro*	transfection, preparation of membranes and binding assays with CB1/CB2 agonists, competition binding assays, cAMP inhibition assays, whole-cell metabolism assay, immunoblot, ESI-LC/MS	Human CB_2_	(Snider et al., 2009)
C57Bl/6 mice, TRPA1-deficient mice (B6129PF2/J), DRG cultures	Mechanical allodynia		*In vivo*	licking time, dynamic plantar and hargreaves behavioral studies, injection of 5,6-EET in hindpaw, Ca-imaging, LC-MS/MS, patch-clamp recordings	TRPA1	(Sisignano et al., 2012)
HEK-293 cells,	pronociceptive		*In vitro*,	Ca-imaging, patch-clamp	TRPV4	(Watanabe et al., 2003)
**8,9-EET**						
human endothelial cell, human coronary artery tissue, vascular smooth muscle cells, bovine aortic endothelial cells	Antiinflammatory	IKκ and NF-κB inhibition	*In vitro*	qPCR, immunohistochemistry (IHC), reversed-phase HPLC, cell-surface enzyme immunoassay, Western Blot, TNFα stimulation	PPARγ	(Node et al., 1999)
DRG neurons, pulmonary murine microvascular endothelial cells		p38 MAPK, ERK pathway	*In vitro, in vivo angiogenesis*	stimulation with EETs/DHETs and quantification with NICI/GC/MS, proliferation assay with [^3^H]thymidine, transwell migration assay, matrigel-based capillary formation assay, Western Blot, qPCR, *in vivo* angiogenesis in subcutaneous sponge model, immunofluorescence of tumors from human non-small cell lung cancer cells		(Obata and Noguchi, 2004; Pozzi et al., 2005)
C57BL/6 WT and sEH KO mice, DRG cultures	Mechanical allodynia	Calcium influx	*In vivo*	injection of lipids or zymosan in hindpaw, Dynamic Plantar behavioral studies, Western Blot analysis, multi-epitope-ligand-cartography (MELC), Ca-Imaging, CGRP-enzyme immunoassay (EIA), LC-MS/MS	TRPA1	(Brenneis et al., 2011)
HUVECs, HCaECs, HaoSMCs, BCAECs, HEK-293, INS-1 832/13		Calcium influx	*In vitro*	transfection of GPCRs, [Ca^2+^] assay with fluorescence, radioligand binding assay, qPCR, immunoblotting, Western Blot, isometric tension measurement, whole-cell patch-clamp	GPR40, GPR120	(Park et al., 2018)
**11,12-EET**						
small bovine coronary arteries, coronary arterial endothelial cells, vascular smooth muscle cells TRVP4 KO mice, second branch order mesenteric resistance arteries	dilation, hyperpolarization	Stimulation of endogenous ADP-ribosylation through G_Sα_, activation of TRPV4-like currents	*in vitro*	vasodilation/-constriction experiments, Patch-clamp electrophysiology	TRPV4, K_Ca_	(Li et al., 1999; Earley et al., 2009)
Sprague-Dawley rats, mononuclear cells		attenuation of PGE_2_	*In vitro*	LPS-stimulation of monocytes, PGE_2_ assay in supernatant of monocytes, Western Blot of COX-2 protein, COX-2 activity assay with [^14^C]AA		(Kozak et al., 2003)
wound tissue of naked SKH-1 mice	Proinflammatory	VEGF and TGFβ expression	*In vivo*	ischemia of the mouse ear, wound creating by circular punch, treatment of wound with different EETs, direct visualization of epithelialization, IHC		(Sommer et al., 2019)
bovine aortic endothelial cells, HUVECs, human coronary artery tissue, vascular smooth muscle cells, Sprague-Dawley rats, RPAECs	Antiinflammatory	Inhibition of p38, degradation IκBα and inhibition of NF-κB, LOX-1 receptor suppression, inhibition of COX-2	*In vitro*	qPCR, IHC, reversed-phase HPLC, cell-surface enzyme immunoassay, TNFα stimulation experiments Immunofluorescence, Cell viability *via* MTT, extraction of cytosolic and nuclear fractions, Western Blot, ELISA		(Node et al., 1999; Liu et al., 2005) (Jiang et al., 2014; Sommer et al., 2019)
HUVECs, HCaECs, HaoSMCs, BCAECs, HEK-293, INS-1 832/13	Proinflammatory	Calcium influx, ERK phosphorylation, COX-2 expression, gab junction, disassembly, cAMP increase	*In vitro, in vivo*	transfection of GPRs, [Ca^2+^] assay with fluorescence, radioligand binding assay, qPCR, immunoblotting, Western Blot, whole-cell patch-clamp	GPR40	(Thompson and Hammock, 2007; Morisseau et al., 2010; Park et al., 2018)
Hippocampus of C57Bl/6 mice	Antinocicpetive	Reduced excitatory transmission	*In vitro*	IHC of brain tissue, electrophysiology	GIRK1/4	(Mule et al., 2017)
**14,15-EET**						
Nude and C57Bl/6 mice, RAW264.7 cells, bone marrow derived macrophages	Antiinflmmatory	NF-κB inhibition, decrease TNFα expression, decrease IL-6,	*in vitro, in vivo cupping therapy*	UPLC-MS/MS, cupping treatment, ELISA, FACS analysis		(Zhang et al., 2018)
porcine thoracic aortic smooth muscle cells (SMC) from SJL mice, brain microvessel, human lung tissue, bronchial ring, Spraque-Dawley rats, BK_Ca_ KO mice, EP3 KO mice, guinea pigs, TRPV4 KO mice, conduit smooth muscle rings and nonvascular smooth muscle strips	Antiinflammatory	Inhibition of PGH synthase, reduction of PGE_2_, vasodilation, inhibition of NF-κB induced transcription	*In vitro*	radiolabelled EETs and AA treatment of cells, revers-phase HPLC, PGE_2_ radioimmunoassay, mRNA analysis by sequential hybridization, Western Blot, DNA synthesis analysis by using [^3^H]thymidine, treatment of bronchial rings with TNFα, EET and AUDA, isometric tension measurements, qPCR, tissue relaxation and contraction experiments, platelet aggregation, competition binding assay, selectivity profile of EET and DHET and human TP receptor and GPRs, ion channels and transporters	TP, EP2	(Fang et al., 1998; Morin et al., 2008; Behm et al., 2009)
mouse mesenteric arteries, HEK-293 cells, follicular membranes of *Xenopus laevis* ooytes			*In vitro*	transfection, cAMP detection assay, oocyte expression system by injection of cRNA, cell surface expression of GPR detection, ERK phosphorylation detection by Western blot luminescence method, PathHunter β-arrestin enzyme fragment complementation assay	EP4, EP2, CXCR4 CMKLR1	(Liu et al., 2017)
Hippocampus of C57Bl/6 mice	Antinociceptive	Reduced excitatory transmission	*In vitro*	IHC of brain tissue, electrophysiology	GIRK1/4	(Mule et al., 2017)
Hippocampus of C57Bl/6 mice		PKA, ERK and CaMKII activation	*In vitro*	electrophysiological recordings of hippocampus, Western Blot analysis		(Wu et al., 2017)
Brain of Sprague-Dawley rats	Antinociceptive	Activation of β-endorphin and met-enkephalin	*In vivo*	Ventrolateral periaqueductal grey matter (vlPAG) microinjection, tail-flick response measurement after radiant heat application, treatment with different drugs, AA or EETs, membrane binding assay of 14,15-EET with μ and δ-opioid receptors		(Terashvili et al., 2008)
Sprague-Dawley rats, primary cortical and sensory neuron culture	Antinociceptive	Axon growth through endogenous 14,15-EET	*In vitro*	Immunocytochemistry, MTT assay of cell viability, morphometric analyses by staining with Protein Gene Product 9.5 or tau-specific antibody, LC-quantification of EETs		(Abdu et al., 2011)
**14,15-DHET**						
HepG2 cells, primary hepatocytes of Sprague-Dawley rats, primary cortical neuron culture	Proinflammatory	Inhibition axon growth	*In vitro*	transactivation assay, liquid chromatography – mass spectrometry (LCMS), gel shift assay, qPCR, immunocytochemistry, MTT assay of cell viability, morphometric analyses by staining with Protein Gene Product 9.5 or tau-specific antibody, LC-quantification of EET	PPARα and γ	(Ng et al., 2007; Abdu et al., 2011)

CaMKII, Ca^2+^/calmodulin-dependent protein kinase II; cAMP, cytosolic adenosine monophosphate; Ca_v_3, T-type calcium channel 3; CCL, chemokine ligand; COX, cyclooxygenase; cRNA, Complementary RNA; DHET, Dihydroxyeicosatrienoic acid; DRG, dorsal root ganglion; EET, epoxyeicosatrienoic acid; ELISA, enzyme-linked immunosorbent assay; EP, prostaglandin E receptor; ER, endoplasmatic reticulum; ERK, extracellular-signal regulated kinase; ESI-LC/MS, electrospray ionization interface for liquid chromatography – mass spectrometry; FACS, fluorescence-activated cell sorting; GIRK, G-protein coupled inwardly rectifying potassium channel; GPR, GPCR, G protein-coupled receptor; HPLC, high performance liquid chromatography; IHC, immunohistochemistry; IKκ, inhibitor of nuclear factor kappa-B kinase; IL, interleukin; KO, knock-out; LC-MS, liquid chromatography–mass spectrometry; LOX, lipoxygenase; LPS, lipopolysaccharide; MAPK, mitogen-activated protein kinases; MELC, multi-epitope-ligand-cartography; MTT, 3(4,5-dimethylthiazol-2-yl)-2,5-diphenyltetrazolium bromide; NF-κB, nuclear factor ‘kappa-light-chain-enhancer’; NICI/GC/MS, negative ion chemical ionization gas chromatography – mass spectrometry; NO, Nitric oxide; PG, prostaglandin; PI3K/Akt, phosphoinositide 3-kinase/protein kinase B; PKA, protein kinase A; PPAR, peroxisome proliferator-activated receptor; qPCR, quantitative real-time polymerase chain reaction; Refs: references; TGF, transforming growth factor; TNF, tumor necrosis factor; TP, thromboxane receptor; TPPU, N-[1-(1-Oxopropyl)-4-piperidinyl]-N’-[4-(trifluoromethoxy)phenyl]urea; TRPA, transient receptor potential ion channel ankyrin subtype; TRPM, transient receptor potential ion channel melastatin subtype; TRPV, transient receptor potential ion channel vanilloid subtype; UPLC-MS/MS, Ultra-performance liquid chromatography–tandem mass spectrometry; VEGF, vascular endothelial growth factor.

In summary, these observations lead to the conclusion, that 5,6-EET can have pronociceptive and proinflammatory effects. However, EETs were found to bind to the anti-inflammatory nuclear peroxisome proliferator-activated receptor (PPAR) α and γ (Hiesinger et al., 2019). Indeed, the binding of EETs to the PPARγ contributes to an anti-inflammatory effect, but only in the low micromolar range (Liu et al., 2005). This low binding effect raises the question whether other, more potent receptors and pathways for EET signaling exist. In endothelial cells it was observed that 5,6-EET is involved in activation of phosphoinositide 3-kinase/protein kinase B (PI3K/Akt) signaling and proliferation (Pozzi et al., 2005) (**Table 1A**).

Although, the binding of 5,6-EET to a corresponding receptor remains elusive. It is known that AA metabolites of cyclooxygenase act through G protein coupled receptors (GPCR), like E and D prostanoid receptors. The endocannabinoids, which have similarities with AA-metabolites as lipid signal messenger, affect nociception through GPCRs like the cannabinoid receptor CB_1_ and CB_2_ indicating that EETs might act through GPCRs as well (Wagner et al., 2011). At low micromolar concentrations EETs displaced the high affinity ligands of peripheral benzodiazepine receptor (PBR), CB_2_ receptor, neurokinin NK_2_ receptor, and dopamine D_3_ receptor (Inceoglu et al., 2007). Moreover, Snider and colleagues could show that 5,6-EET ethanolamide binds with high affinity to human CB_2_ receptor (Snider et al., 2009) (**Table 1A**). However, a specific 5,6-EET receptor is not known yet.

#### 8,9-EET

EETs enter cells and interact directly with intracellular effectors like fatty acid-binding proteins (FABP) and PPARγ (Widstrom et al., 2001; Widstrom et al., 2003; Fleming, 2007). Thus, EETs increase PPARγ transcription activity, a key molecular event involved in inhibiting NF-kB contributing to the anti-inflammatory effect of the EETs (Liu et al., 2005; Norwood S. et al., 2010). Blocking of PPARγ reduced EET/sEH inhibitor-mediated anti-inflammatory effects (Norwood S. et al., 2010). However, 8,9-EET inhibited NF- kB activation to a lesser extent than 11,12-EET in endothelial cells (Node et al., 1999) (**Table 1A**).

In sEH knock-out mice, 8,9-DHET levels were reduced leading simultaneously to an increased 8,9-EET level. Thereby, 8,9-EET increases intracellular calcium concentrations in cultured DRG neurons (Brenneis et al., 2011). The calcium increase induced the activation of mitogen-activated protein kinase (MAPK) p38 and extracellular-signal regulated kinase (ERK) most potently with 8,9-EET, resulting in increased nociceptive activity (Obata and Noguchi, 2004; Pozzi et al., 2005). In this context, it was shown that 8,9-EET can sensitize TRPA1 expressing primary afferent neurons and may contribute to a reduced mechanical pain threshold (Brenneis et al., 2011) (**Table 1A**). However, one only can speculate about a direct interaction of 8,9-EET with TRPA1.

Additionally, it was shown that EETs activate translocator protein (TSPO) and increase steroidogenic acute regulatory protein 1 (StARD1) expression in the spinal cord resulting in an upregulation of circulating progesterone, an analgesic molecule and precursor for neurosteroid production (Inceoglu et al., 2008). How this activation of TSPO is mediated is currently unknown. One possible way could be through the activation of a GPCR. Stimulation of the free fatty acid receptor-1 (FFAR1, also GPR40) with 8,9-EET leads to an increase in intracellular calcium concentration in FFAR1 and GPR120 expressing cells. However, in comparison to the other regioisomers, 5,6- and 8,9-EET were less potent for FFAR1 stimulation. In contrast, the receptor for long chain ω-3 fatty acids GPR120, was stimulated equally by 8,9-, 11,12- and 14,15-EET (Park et al., 2018) (**Table 1A**).

Furthermore 11,12-EETs activates K^+^_Ca_ channels in coronary smooth muscle cells *via* a G_αs_-mediated mechanism (Li and Campbell, 1997). Since the structure of 11,12-EET is very similar to that of 8,9-EET, 8,9-EET might act in a same signaling manner and may be able to activate the unidentified EET-receptor in a Gα-mediated mechanism.

#### 11,12-EET

The role of 11,12-EET in activation of different K^+^ channels, especially in the cardiovascular milieu has been extensively studied (Krotz et al., 2004; Lu et al., 2006). The occurring activation *via* the G_αs_ receptor leads to hyperpolarization and arterial dilation through TRPV4 activation (Li et al., 1999; Earley et al., 2009). However, 11,12-EET does not have any effect on neuronal TRPV4 channels (Brenneis et al., 2011). Earlier studies indicate that levels of 11,12-EET are not altered in any tissue or nociceptive model and peripheral injection of 11,12-EET did not cause any nocifensive behavior in mice (Brenneis et al., 2011; Sisignano et al., 2012) (**Table 1A**).

Moreover, the role of 11,12-EET in the context of inflammation has been studied thoroughly. The 11,12-EET was found capable of inhibiting NF-kB after LPS-induction or TNFα treatment at nanomolar concentrations (Node et al., 1999; Liu et al., 2005). LPS-stimulated cells treated with 11,12-EET showed decreased prostaglandin E_2_ (PGE_2_) secretion by inhibition of COX-2 activity (Kozak et al., 2003). This contributes further to the anti-inflammatory effect of 11,12-EET as well as the inhibition of p38 phosphorylation, the degradation of IκBα and the suppression of LOX-1 receptor expression (Node et al., 1999; Jiang et al., 2014). In wounds after ischemia, 11,12-EET increased both vascular endothelial growth factor (VEGF) and transforming growth factor (TGF)-ß expression leading to improved wound healing (Sommer et al., 2019). In inflammatory pain, exogenous delivery of different epoxides or the stabilization of EETs *via* inhibition of soluble epoxide hydrolase (sEH) in the paw resulted in reduced mechanical pain hypersensitivity *in vivo* (Inceoglu et al., 2007; Morisseau et al., 2010) (**Table 1A**). However, in this study, all four EET regioisomers were administered, and it remains unclear, which of the EETs is the most active compound in this context.

In contrast, activation of free fatty acid receptor-1 (FFAR1, also GPR40) through 11,12-EET leads to increased COX-2 expression, NF-κB activation and ERK phosphorylation resulting in gap junction disassembly (Popp et al., 2002; Michaelis et al., 2005; Park et al., 2018). The activation of FFAR1 was mediated by a G_q_ signaling pathway to increase the intracellular calcium concentration and cyclic adenosine monophosphate (cAMP) (Hauge et al., 2015; Park et al., 2018). Decreased levels of cAMP were observed with the diol 11,12-DHET suggesting the involvement of a G_αi_ signaling pathway (Abukhashim et al., 2011). However, there is a lot more evidence for 11,12-EET acting through a G_S_ signaling mechanism (Node et al., 2001; Ding et al., 2014; Hauge et al., 2015). Interestingly, EET effects were not altered by FFAR1 antagonism indicating the existence of an 11,12-EET receptor that compensates for the reduced FFAR1 activation. However, Park et al. suggested a direct interaction of 11,12-EET with FFAR1, which might be the receptor for tans-EETs as it is for trans-fatty acids (Park et al., 2018). In this study the corresponding diols, which are thought to be inactive products of EETs, showed less stimulatory activity on FFAR1 than the parent EETs (Spector et al., 2004; Park et al., 2018).

Nevertheless, binding to GPCRs remains controversial. Recently 11,12-EET and -DHET were reported to bind to the GPR132 (G2A). Interestingly, 11,12-DHET was the more potent activator of G2A, even if it is still a low affinity receptor with physiological relevance in hematopoiesis (Lahvic et al., 2018). In this regard, 11,12-DHET seems to be a critical modulator of progenitor cell proliferation and mobilization by activation of the canonical int-1 and related genes (Wnt) signaling cascade (Fromel et al., 2012) (**Table 1A**).

In the neuronal system, 11,12-EET opens G protein-coupled inward rectifier potassium (GIRK) channels in CA1 pyramidal cells in mouse hippocampus *via* G_i/o_ proteins (Mule et al., 2017). This leads to reduced excitatory transmission in hippocampus acting on pre- and postsynaptic targets (Mule et al., 2017). However, in cultured DRG neurons, 11,12-EET had no effect on the intracellular calcium concentration, since it may inhibit L-type Ca^2+^-channels independent of intracellular cAMP levels or protein phosphorylation (Chen et al., 1999). The intracellular cAMP concentration plays an important role during the increase of transient receptor potential ion channel canonical subtype (TPRC)6 channel translocation to the plasma membrane; an effect mediated by 11,12-EET (Fleming et al., 2007; Loot and Fleming, 2011; Ding et al., 2014) (**Table 1A**).

A common approach to increase EET levels is to use either an activator of CYP epoxygenases, like omeprazole, or a sEH inhibitor like, for example, 1-Trifluoromethoxyphenyl-3-(1-propionylpiperidin-4-yl) urea (TPPU). This leads to a simultaneous decrease of DHET and DiHOME levels in plasma and to enhanced anti-hyperalgesia in an acute inflammatory pain model (Inceoglu et al., 2006; Goswami et al., 2015). However, this effect might not only be due to 11,12-EET and may rather be caused by the mixture of EETs, because 11,12-EET alone has been shown to have no effect on nociceptive behavior in acute pain models (Sisignano et al., 2012).

In summary, 11,12-EET plays important roles in the cardiovascular system and during inflammation. But, its individual role in peripheral neurons and nociceptive behavior seems to be negligible.

#### 14,15-EET

The effects of 14,15-EET are widely studied in the cardiovascular milieu. In this context it reduced proteasomal and caspase-3 activity having anti-apoptotic effects and by that promoting the survival of cardiac cells (Chen et al., 2001; Samokhvalov et al., 2013).

In an inflammatory context 14,15-EET can cause activation of PPARγ leading to a decreased NF-κB response and TNFα expression in cardiomyocytes (Liu et al., 2005; Samokhvalov et al., 2014). Even after cupping therapy, IL-6 and TNFα were decreased (Zhang et al., 2018). Surprisingly, the diol 14,15-DHET is together with 11,12-EET the most potent activator of PPARγ and PPARα (Ng et al., 2007) (**Table 1A**).

In later time points of acute zymosan-induced inflammation 14,15-EET levels were decreased (Brenneis et al., 2011). They were increased when sEH was deleted, but did not alter the zymosan-induced inflammatory hyperalgesia by inhibiting the prostaglandin synthesis (Brenneis et al., 2011; Zhang et al., 2014). The inhibition was also observed in smooth muscle cells, where 14,15-EET acts as competitive inhibitor of prostaglandin H (PGH) synthase and thus reduce PGE_2_ reduction (Fang et al., 1998). Anti-inflammatory effects were also observed in human bronchi with 14,15-EET after TNFα stimulation (Mule et al., 2017). These effects might occur due to 14,15-EET antagonizing the thromboxane receptor (Morin et al., 2008; Behm et al., 2009). In contrast, the anti-inflammatory effect was missing in bovine aortic endothelial cells after treatment with 14,15-EET and TNFα (Node et al., 1999). In conclusion, the anti-inflammatory effects of 14,15-EET depend on the organism and the inflammation context and seem to be weaker, as with 11,12-EET.

The actions of 14,15-EET are connected to Gαs signaling increasing the expression of tissue-type plasminogen activator followed by an increase of intracellular cAMP (Node et al., 2001). The elevated levels of cAMP then act in a negative feedback loop and decrease binding of 14,15-EET (Wong et al., 2000).

The effects of 14,15-EET may be mediated by a receptor. So far, there are various receptors known which might act as 14,15-EET receptor. In a screen of GPCRs, Liu and colleagues identified as putative 14,15-EET receptors the prostaglandin receptor subtypes EP_4_ and EP_2_. The EP_2_ receptor is postulated to mediate the vasodilatory effect of 14,15-EET and not as 11,12-EET does, through the activation of BK_Ca_ channels or TRPV4 (Wong et al., 1997; Yang et al., 2008; Behm et al., 2009; Earley et al., 2009; Spector and Kim, 2015). However, very little dilatatory effects were seen *in vivo* in cerebral arterioles with 14,15-EET (Ellis et al., 1990).

Another GPCR, the free-fatty acid receptor FFAR1 (also known as GPR40), was found to be activated by 14,15-EET in a similar potency as with 11,12-EET. Whereas 11,12-DHET and 14,15-DHET were less active in stimulating FFAR1 (Park et al., 2018).

The GPCR G2A was activated by 14,15-EET as well, but only the zebrafish GPR132 and not the human variant (Lahvic et al., 2018).

Additionally, C-X-C chemokine receptor type 4 (CXCR4) and Chemerin chemokine-like receptor 1 (CMKLR1) receptors were activated by 14,15-EET. However, none of these receptors meet the criteria of a high-affinity receptor for 14,15-EET (Liu et al., 2017). Thus, it is still unclear whether 14,15-EET is a natural ligand of one of the named receptors or whether there is a high-affinity receptor, which is still unknown.

Regarding nociception, 14,15-EET had no effect on nociceptive behavior in mice after injection into the paw (Brenneis et al., 2011), but it was capable of reducing thermally induced pain in a dose-dependent manner (Terashvili et al., 2008). The anti-nociceptive effect of 14,15-EET was also observed after injection into the rat ventrolaterale periaqueductal gray due to the activation of ß-endorphin and met-enkephalin (Terashvili et al., 2008). Similar results were obtained with morphine indicating an endogenous opioid dependent analgesia (Terashvili et al., 2008). Additionally, neurons are able to produce endogenous 14,15-EET leading to stimulated axon growth in primary cortical and sensory neuron cultures (Abdu et al., 2011). In contrast, its corresponding diol, 14,15-DHET seems to inhibit axonal growth (Abdu et al., 2011) (**Table 1A**).

In summary, all four regioisomers have different but overlapping effects. The 5,6-, 8,9- and 14,15-EET can act through PPARγ (Liu et al., 2005; Pober and Sessa, 2014). However, the underlying pathways seem to be different. The 5,6-EET can activate the PI3A/Akt pathway and has vasodilatatory effects on cerebral arterioles, whereas the 14,15-EET has almost no dilatatory effect in this setting (Pozzi et al., 2005; Pober and Sessa, 2014). Regarding neuropathic pain and nociceptive behavior, there is only little known about their role. The 5,6- and 8,9-EET induce acute pain behavior through activation and/or sensitization of TRPA1 in peripheral sensory neurons (Brenneis et al., 2011; Sisignano et al., 2012). In contrast, 11,12- and 14,15-EET showed no direct effects on nociceptive behavior (Brenneis et al., 2011; Sisignano et al., 2012). Only 14,15-EET seems to have anti-nociceptive effects and seems to play a role in the excitatory transmission in the peripheral and central nervous system (Terashvili et al., 2008). Thus, all EETs seem to act *via* individual mechanisms and pathways, which should be kept in mind when using sEH inhibitors as potential therapeutic agent, since it affects all the regioisomers and thus can have different effects at the same time in the whole organism.

There is large evidence that EETs can activate a G_αs_-coupled GPCR (Li et al., 1999). However, a specific EET-receptor remains unknown.

### EpOMEs and DiHOMEs

Next to the AA metabolites, LA products have effects on nociception and pain as well. 9,10- and 12,13- epoxyoctadecenoic acid (EpOME) are some of those metabolites, synthesized by epoxidation of LA (Oliw, 1994). They are also called leukotoxins, because EpOMEs are produced after treatment with LA and calcium by inflammatory leukocytes such as macrophages and neutrophils (Hayakawa et al., 1986; Thompson and Hammock, 2007). Neutrophil influx with increased production of EpOME levels occur for example in acute respiratory distress syndrome (Ozawa et al., 1988). When neutrophils are treated with EpOMEs a slight respiratory burst occurs, which was strongly inhibited by the corresponding diols 9,10- and 12,13-DiHOME, generated by soluble epoxide hydrolase (Inceoglu et al., 2006; Thompson and Hammock, 2007; Morisseau et al., 2010). Interestingly, DiHOMEs showed more toxic effects than the EpOMEs in acute respiratory distress syndrome (Zheng et al., 2001). Similar results were observed in endothelial cells, where DiHOMEs disrupted endothelial barrier function and induced more IL-6 expression than the EpOMEs (Moghaddam et al., 1997; Greene et al., 2000a; Slim et al., 2001). A detoxification pathway by glucuronidation of the diols was postulated by Greene and colleagues (Greene et al., 2000a). On the other hand, the diols were incorporated into phospholipids and therefore the toxicity of free diols was lost (**Table 1B**).

**Table 1B:** Metabolites of linoleic acid (LA) in inflammation and pain.

Animals/tissue/cell type	Effect	Signaling	Experiment	Experimental details	Target	Refs.
9,10-/12,13-DiHOME						
porcine pulmonary artery endothelial cells, SF-21 cells, alveolar type II cells, Sprague-Dawley rats, Swiss-Webster mice	Proinflammatory	IL-6 expression, disruption endothelial barrier function	*In vitro, in vivo*	chemical synthesis of leukotoxins, baculovirus expression system, TLC, GC, GC/EI/MS, NMR, heart puncture with free fatty acids or tail vein injections of free fatty acids, endothelial barrier function measurement by transendothelial albumin transfer with bromcresol green, electrophoretic mobility shift assays with nuclear extracts, RT-PCR, measurement of epoxide hydrolase activity with tDPPO, fatty acid analysis by gas chromatography, metabolite studies via liquid scintillation counter (LSC) and radioactivity measurement, PLA2 hydrolysis measurement, esterase assays, glutathione assay and conjugate formation		(Moghaddam et al., 1997; Greene et al., 2000a; Slim et al., 2001)
*Ephx2*-gene deficient mice, HL-1 cardiac cells, neonatal rat cardiomyocytes	Proinflammatory	Release of TNFα and MCP-1	*In vitro*	mitochondrial function *via* citrate synthase, NADH:ubiquinone oxidoreductase and succinate dehydrogenase activity measurement, mitochondrial respiration by glutamate and ADP-stimulated respiration, immunoblotting, aconitase activity, 20S proteasome and malondialdehyde assays, LC-MS/MS, echocardiography, ELISA of TNFα and MCP-1, treatment of cells with different EpOMEs, DiHOMEs, ATP-level measurement *via* luciferase-based method		(Slim et al., 2001; Samokhvalov et al., 2018)
Neutrophils, Sprague-Dawley rats, blood, HL-60 cells	Proinflammatory	Respiratory burst	*In vivo, in vitro*	Hargreaves radiant heat and von Frey filament behavioral studies, EETs or sEH inhibitors applied *via* cream, reverse-phase HPLC, LC-MS/MS, WST-1-reducing activity measurement by lucigenin-dependent chemiluminescence, β-glucuronidase release assay, immunoblot analysis, Western Blot		(Inceoglu et al., 2006; Thompson and Hammock, 2007; Morisseau et al., 2010)
Lung tissue of Swiss Webster mice	Proinflammatory	Respiratory distress syndrome	*In vivo*	histopathology *via* inflation with fixation *via* a tracheal cannula, immersion deflated or immersion after maintenance of inflation by ligation of the trachea, synthesis of leukotoxin and gas liquid chromatography, sEH preparation of liver cytosol, immunocytochemistry		(Zheng et al., 2001)
*Ephx2*-gene deficient mice, HL-1 cardiac cells, neonatal rat cardiomyocytes, HepG2 cells	Proinflammatory	Reduced cell viability, attenuation of insulin signal, collapse in mitochondrial function, ER stress, cell death	*In vitro, in vivo*	mitochondrial function *via* citrate synthase, NADH:ubiquinone oxidoreductase and succinate dehydrogenase activity measurement, mitochondrial respiration by glutamate and ADP-stimulated respiration, immunoblotting, aconitase activity, 20S proteasome and malondialdehyde assays, LC-MS/MS, echocardiography, ELISA of TNFα and MCP-1, treatment of cells with different EpOMEs, DiHOMEs, ATP-level measurement *via* luciferase-based method, High fat diet of mice, treatment with sEH-inhibitor, Western Blot, qPCR		(Bettaieb et al., 2013; Samokhvalov et al., 2018)
Plasma, female participants age 20-65 y, C57BL/6N WT and TRPV1 KO mice, DRG cultures	Thermal hypersensitivity	Decreased amount, TRPV1 sensitization	*Clinical, in vivo*	Blood sample lipid extraction and analysis with UPLC, zymosan/CFA/lipid injection in hind paw, oral administration of TPPU, thermal behavioral studies, LC-MS/MS, Ca-imaging, CGRP ELISA,		(Hellstrom et al., 2016; Zimmer et al., 2018)
C57BL/6N WT and TRPV1 KO mice, DRG cultures	Thermal hyperalgesia	Ca-transient induction in sensory neurons	*In vivo, in vitro*	zymosan/CFA/lipid injection in hind paw, oral administration of TPPU, Hargreaves radiant heat behavioral studies, LC-MS/MS, Ca-imaging, CGRP ELISA		(Zimmer et al., 2018)
**EpOME**						
Sprague-Dawley rats, CHO-cells, C57Bl/6 mice, TRPV1 KO mice, primary DRG cultures	Proinflammatory, Pronociceptive	TRPV1 sensitization, calcium influx, PKA-activation	*In vitro, in vivo*	peripheral burn injury, paclitaxel-induced neuropathic pain, paw-withdrawal latency measurement with radiant heat and dynamic plantar, drug injection, HPLC-ESI-MS/MS, transfection, calcium imaging on sensory neurons, electrophysiology, blood pressure measurements, qPCR, LC-MS/MS, [^35^S]GTPγS binding assays, peripheral burn injury	TRPA1, TRPV1	(Green et al., 2016; Sisignano et al., 2016; Zimmer et al., 2018)
**13-HODE**						
Mongrel dogs heart arteries, canine splenic artery, porcine SMCs	Proinflammatory	Relaxation of coronary arteries, calcium influx, cGMP level increase, stimulation of biosynthesis of COX-derived vasodilators	*In vitro*	vascular reactivity measurements, NO release assay, biosynthesis of prostacyclin *via* radioimmuoassay, HPLC analysis of 13-HPODE and 13-HODE, Ca-imaging, cGMP levels by radioimmunoassay	L-type channels, TP	(De Meyer et al., 1992; Stoll et al., 1994)
RAW264.5 (ATCC) cells, peritoneal macrophages			*In vitro*	western blot analysis, northern blot analysis, transient transfection	PPARγ	(Ricote et al., 2000)
HEK293, rat DRG cells	Cold sensitivity	Antagonizing TRPM8-mediated calcium influx	*In vitro*	TRPV1/TRPV2/TRPA1/TRPM8 transfection, Ca-imaging		(De Petrocellis et al., 2012)
HEK293, rat DRG cells, C57Bl/6 WT and TRPV1 KO mice, Sprague-Dawley rats; CHO cells; TG cultures G2A KO mice, DRG cultures,	Mechanical allodynia, thermal hyperalgesia	TRPV1-sensitization	*In vitro, in vivo*	TRPV1/TRPV2/TRPA1/TRPM8 transfection, Ca-imaging, HPLC and MS analysis, electrophysiology, immunoreactive CGRP (iCGRP) release, Oxaliplatin-treatment, 9-HODE injection in hind paw, mechanical and thermal behavioral studies, LC-MS/MS, Ca-imaging, CGRP ELISA, electrophysiology, qPCR, lipophilic substance isolation from superfusate of spinal cord tissue, electrophysiology, 9-HODE ELISA, CFA treatment, λ-Carrageenan injection into paw and 15-LOX inhibitor and anti-13- and -9-HODE injection, hyperalgesia measurements, LC-MS/MS		(Patwardhan et al., 2009; Patwardhan et al., 2010; De Petrocellis et al., 2012; Alsalem et al., 2013; Hohmann et al., 2017)
**9-HODE**						
THP-1, HeLa, CV-1 cells, human peripheral blood, Cos7 cells, human peripheral monocytes, patients with type 2 diabetes		Macrophage gene expression during atherogenesis, increase of FABP4 expression	*In vitro*, clinical	northern analysis, native and oxidized LDL association and uptake *via* labelled LDL or oxLDL measurements with flow cytometry, transfection, ligand binding assays, protein expression and purification, crystallisation, structure determination, ligand assignment, mass spectrometry, circular dichroism of thermal denaturation measurements, transfection and reporter gene assay, incubation of cells with LA, 9- or 13-HODE and/or with PPARγ antagonist, multiplex flowcytomix system for cytokine measurement, flow cytometry of activated monocytes, immunocytochemistry, qPCR, GPR132 gene silencing with siRNA	PPARγ	(Nagy et al., 1998; Ricote et al., 2000; Itoh et al., 2008; Vangaveti et al., 2018)
CHO-K1 cells, HEK-293 cells	Proinflammatory	IL-6, IL-8 and GM-CSF release, PKC-dependent TRPV1 sensitization	*In vitro*	transfection, flow cytometry, RT-PCR, Ca-imaging, measurement of intracellular cAMP concentration with cAMP-Screen System, GTPγS binding assay, Western Blot, *in vitro* peroxidation, anion-exchange chromatography and measurement of radioactivity	G2A	(Obinata et al., 2005; Kabarowski, 2009)
female participants age 20-65 y		Elevated plasma levels	Clinical	Blood sample lipid extraction and analysis with UPLC		(Hellstrom et al., 2016)
HEK293, rat DRG cells, C57Bl/6 WT and TRPV1 KO mice, Sprague-Dawley rats; CHO cells; TG cultures G2A KO mice, DRG cultures,	Mechanical allodynia, thermal hyperalgesia, OPIN	TRPV1-sensitization	*In vitro, in vivo*	TRPV1/TRPV2/TRPA1/TRPM8 transfection, Ca-imaging, HPLC and MS analysis, electrophysiology, CGRP release, Oxaliplatin-treatment, 9-HODE injection in hind paw, mechanical and thermal behavioral studies, LC-MS/MS, Ca-imaging, CGRP ELISA, electrophysiology, qPCR, lipophilic substance isolation from superfusate of spinal cord tissue, electrophysiology, 9-HODE ELISA, CFA treatment, λ-Carrageenan injection into paw and 15-LOX inhibitor and anti-13- and -9-HODE injection, LC-MS/MS		(Patwardhan et al., 2009; Patwardhan et al., 2010; De Petrocellis et al., 2012; Alsalem et al., 2013; Hohmann et al., 2017)
**13H-9,10E-LA**						
Sprague-Dawley rats, hind paw, sciatic nerve, DRG, trigeminal ganglia (TG), dorsal horn tissue, human skin biopsies	Proinflammatory	Sensitization of afferent DRG neurons, thermal hypersensitivity	Clinical	fatty acid analysis, gas chromatography, RNAseq, LC-MS/MS, CGRP release assays, intradermal injection of lipids in mice, scratching and thermal behavioral studies,		(Ramsden et al., 2017)
C57Bl/6 mice, brain tissue	Chronic inflammation	Reduced amounts	*In vivo*	CFA treatment of ankles, solid phase extraction following LC-MS/MS		(Jensen et al., 2018)
**11H-12,13E-LA**						
C57Bl/6 mice, brain tissue (Amygdala, PGA), Sprague-Dawley rats, hind paw, sciatic nerve, DRG, TG, dorsal horn tissue, human skin biopsies	Proinflammatory, pronociceptive	Sensitization of afferent DRG neurons, headaches	Clinical, *in vitro, in vivo*	CFA treatment of ankles, solid phase extraction following LC-MS/MS, fatty acid analysis, gas chromatography, RNAseq, LC-MS/MS, CGRP release assays, intradermal injection of lipids in mice, scratching and thermal behavioral studies		(Ramsden et al., 2017; Jensen et al., 2018)
**9K-12,13E-LA**						
Sprague-Dawley rats, hind paw, sciatic nerve, DRG, TG, dorsal horn tissue, human skin biopsies	Proinflammatory	itch	Clinical, *in vitro, in vivo*	fatty acid analysis, gas chromatography, RNAseq, LC-MS/MS, CGRP release assays, intradermal injection of lipids in mice, scratching behavior, thermal behavioral studies,		(Ramsden et al., 2017)

13-/9-HODE,13-/9-hydroxyoctadecadienoic acid; 13H-9,10E-LA,13-hydroxy-9,10-trans-epoxy-octadecenoate; 11H-12,13E-LA, 11-hydroxy-12,13-trans-epoxy-(9Z)-octadecenoate; 9K-12,13E-LA, 9-keto-12,13-trans-epoxy-10E-octadecenoate; cAMP, cytosolic adenosine monophosphate; CFA, complete Freund’s adjuvant; cGMP, cyclic guanosine monophosphate; CGRP, calcitonin gene-related peptide DiHOME, dihydroxyoctadecenoic acid; EpOME, epoxyoctadecenoic acid; G2A, G2 accumulation GPCR; GPR, GPCR, G protein-coupled receptor; GC/EI/MS, gas chromatography electron ionisation mass spectrometry; GM-CSF, granulocyte-macrophage colony-stimulating factor; IL, interleukin; KO, knock-out; LC-MS/MS, liquid chromatography tandem mass spectrometry LDL, low-density lipoprotein; MCP, monocyte chemoattractant protein; NADH, nicotinamide adenine dinucleotide; NMR, nuclear magnetic resonance; NO, nitric oxide; PKC, protein kinase C; PLA, phospholipase A; PPAR, peroxisome proliferator-activated receptor; Refs, references; tDPPO, trans-diphenylpropene oxide; sEH, soluble epoxide hydrolase; TG, trigeminal ganglia; TLC, thin layer chromatography; TPPU, N-[1-(1-Oxopropyl)-4-piperidinyl]-N’-[4-(trifluoromethoxy)phenyl]urea; WT, wild-type.

This toxicity of DiHOMEs caused a reduced cell viability, attenuation of insulin signal, overall collapse in mitochondrial function and endoplasmatic reticulum stress as well as cell death in murine hearts (Bettaieb et al., 2013; Samokhvalov et al., 2018). Furthermore, DiHOMEs seem to promote proinflammatory cascades such as NF-κB because of a massive release of TNFα and Monocyte chemoattractant protein 1 (MCP-1) from HL-1 cells (Slim et al., 2001; Samokhvalov et al., 2018).

Thus, in acute and persistent inflammatory pain mouse models application of 12,13-DiHOME leads to increased thermal hypersensitivity due to its sensitizing effects on TRPV1, whereas 9,10-DiHOME showed no effect (Inceoglu et al., 2006; Eskander et al., 2015; Zimmer et al., 2018). Interestingly, in chronic inflammatory pain reduced concentrations of EpOMEs and 9,10-DiHOME were observed in the amygdala and the periaqueductal gray (Jensen et al., 2018).

In patients with chronic neck pain, plasma levels of 9,10- and 12,13-DiHOME were increased, although a direct correlation between the elevated metabolites and pain levels is missing (Hellstrom et al., 2016).

Several more pain models were investigated concerning the effects of EpOMEs and DiHOMEs on the different pain states. In a model of peripheral burn injury both mediators were increased in spinal cord and caused activation of TRPV1 and TRPA1 in heterologous expression systems, suggesting TRPV1- and TRPA1-mediated effects on mechanical and thermal hypersensitivity (Green et al., 2016). However, in DRG cultures 12,13-DiHOME showed no effect on TRPA1 sensitization, indicating TRPA1 sensitization through 9,10-DiHOME (Zimmer et al., 2018) (**Table 1B**).

Regarding chemotherapy-induced neuropathic pain, levels of 9,10-DiHOME and 12,13-DiHOME were unaltered, whereas 9,10-EpOME was increased in DRG neurons (Sisignano et al., 2016; Hohmann et al., 2017). It was synthesized by neurons and glial cells and contributed to calcium influx in DRG neurons due to a protein kinase A (PKA)-mediated sensitizing effect on TRPV1 (Sisignano et al., 2016). As a result, thermal and mechanical hypersensitivity was elevated in paclitaxel-induced neuropathic pain *via* 9,10-EpOME (Sisignano et al., 2016).

Thus, it seems that DiHOMEs and EpOMEs have different effects on the various kinds of neuropathic pain. How those effects are mediated by the metabolites is not examined yet. It is still unclear, whether there is a receptor for EpOMEs or DiHOMEs. For the G2A receptor a low affinity binding of EpOMEs has been reported (Lahvic et al., 2018). Regarding the described high toxicity of DiHOMEs Greene and colleagues proposed that it does not seem to be a receptor-mediated mechanism (Greene et al., 2000b).

### HODEs

The hydroperoxides HODEs are stable oxidation products of LA. Their concentrations are elevated under oxidative stress such as ischemic brain injury or cardiac arrest (Liu et al., 1998; Yoshida and Niki, 2006; Hennebelle et al., 2017). How the HODEs are synthesized is not fully understood. An earlier study suggested that 15-LOX oxidized LA to 13-HPODE, that is reduced through glutathione–peroxidase to 13-HODE (Reinaud et al., 1989). Inhibition experiments indicate that especially 13-HODE is produced by LOX enzymes, whereas, 8-,9-, 11- and 14-HODE are produced in microsomes either through PGH synthase, lipoxygenase or non-enzymatically (Oliw, 1994; Engels et al., 1986; Green, 1989; De Meyer et al., 1992; Jisaka et al., 1997).

The 13-HODE increases calcium influx and intracellular cGMP levels inducing relaxation of canine splenic and coronary artery segments (De Meyer et al., 1992; Stoll et al., 1994). This effect might be mediated through an L-type channel that is regulated by a cGMP-dependent kinase (Stoll et al., 1994). On the other hand, at high concentrations 13-HODE contract the segments by activation of thromboxane receptor (De Meyer et al., 1992). Together with 9-HODE, it also binds in a micromolar range to PPARγ, depending on the activation state of macrophages, and causes increased activity of fatty acid binding protein 4 (Nagy et al., 1998; Ricote et al., 2000; Itoh et al., 2008; Vangaveti et al., 2018) (**Table 1B**). A receptor with higher binding affinity for 13-HODE has not yet been identified.

However, 9(S)- and 13-HODE seem to antagonize intracellular calcium influx, mediated by the cold-thermosensor of sensory nerves in deeper tissue TRPM8 and seem to activate TRPA1 in a heterologous expression system. However, 13-HODE was more potent in TRPA1 activation than 9-HODE (De Petrocellis et al., 2012). In this system, 9-HODE was also able to activate TRPV1-mediated intracellular calcium elevation at a nanomolar range, whereas 13-HODE was not such effective (Patwardhan et al., 2009; Patwardhan et al., 2010; De Petrocellis et al., 2012; Alsalem et al., 2013). In trigeminal neurons, capsaicin-sensitive neurons responded to 9-HODE by an increase of intracellular calcium level (Patwardhan et al., 2009; Patwardhan et al., 2010). Injection of 9-HODE and 13-HODE into the paw caused strong spontaneous nociceptive behavior and thermal hypersensitivity in rats (Patwardhan et al., 2010). Interestingly, when 9-HODE was injected intrathecally a longer mechanical allodynia was seen than with capsaicin (Patwardhan et al., 2009). Antinociception and reversion of mechanical allodynia was obtained when LA oxidation or HODEs were blocked (Patwardhan et al., 2009; Patwardhan et al., 2010). However, intrathecal injection of 9- and 13-HODE blocking antibodies had no effect on thermal allodynia (Green et al., 2016). In the periphery, the concentrations of 9- and 13-HODE as well as their metabolites 9- and 13-oxoHODE were elevated after severe burn injury, in irradiated or in heated skin and mediated inflammatory heat hyperalgesia (Patwardhan et al., 2010; Alsalem et al., 2013; Green et al., 2013; Sisignano et al., 2013). The occurring thermal hypersensitivity was reduced by injection of 9- and 13-HODE blocking antibodies into the paw, suggesting a local effect of the LA metabolites. This local effect of 9- and 13-HODE blocking antibodies was also seen in thermal injury and in chronic inflammatory hyperalgesia in the paw, as well as after inhibition of CYP enzymes, indicating CYP enzymes to produce TRPV1 active metabolites of LA (Ruparel et al., 2012a; Green et al., 2013). Likewise, an attenuation of inflammatory hyperalgesia was observed after inhibition of 15-LOX in paw tissue, but no altered calcium response was produced by exogenous 9- and 13-HODE in DRGs. The authors concluded both lipids to be responsible for the TRPV1-mediated calcium influx (Alsalem et al., 2013).

In chemotherapy-induced neuropathic pain 9-HODE levels were elevated in sciatic nerve and DRG neurons indicating a release by neuronal tissue (Hohmann et al., 2017). Both, 9- and 13-HODE also can be generated from non-neuronal tissue (Patwardhan et al., 2010) (**Table 1B**). However, the concrete binding action to a receptor of 13-HODE remains elusive.

In contrast, 9-HODE binds to the GPCR G2A, a receptor that is expressed by immune cells, especially by macrophages and T cells (Obinata et al., 2005; Kabarowski, 2009), but also by TRPV1-positive primary sensory neurons (Kwan et al., 2007). In contrast, 13-HODE only showed moderate activity on G2A (Yin et al., 2009; Hohmann et al., 2017).

The 9-HODE evokes intracellular calcium mobilization, activation of c-Jun N terminal kinase (JNK), and inhibition of cAMP accumulation (Obinata et al., 2005). In human keratinocytes and HaCaT cells oxidative stress resulted in G2A expression and 9-HODE generation. The 9(S)-HODE then caused enhanced IL-6, -8 and release of granulocyte-macrophage colony-stimulating factor (GM-CSF), as well as inhibited proliferation due to cell cycle arrest in the G0/1-phase (Hattori et al., 2008; Obinata and Izumi, 2009). These effects were not observed with 13-HODE (Hattori et al., 2008).

G2A activation by 9-HODE is concentration-dependent and in oxaliplatin-induced peripheral neuropathic pain (OIPN) it can lead to TRPV1 sensitization (Hohmann et al., 2017). Injection of 9-HODE during OIPN causes increased mechanical hypersensitivity *in vivo* that seems to be mediated by a protein kinase C (PKC)-dependent mechanism, whereas direct injection into the paw of naïve wild-type mice had no effect on nociceptive behavior (Hohmann et al., 2017) (**Table 1B**).

In conclusion, 9-HODE plays an important role during inflammatory and neuropathic pain, possibly by binding to the G2A receptor and sensitizing TRPV1. On the other hand, the effects of 13-HODE seem to be more restricted to the skin and the cardiovascular milieu. However, all studies were performed *in vitro* or in mice so far. Less is known about the effects of 9- and 13-HODE in chronic or persistent pain patients. One study observed elevated 9- and 13-HODE plasma levels in patients with chronic neck pain (Hellstrom et al., 2016). Herein, 9- and 13-HODE levels showed a positive correlation to the daily pain rate (Hellstrom et al., 2016). Nevertheless, further investigations are needed to evaluate the role of 9- and 13-HODE in chronic and persistent pain in humans.

### Additional Metabolites of LA and AA

Recently further LA metabolites were found to play a role in pain and itch. In inflamed psoriatic human skin, the concentrations of the previously unknown endogenous lipids 11-hydroxy-12,13-*tans*-epoxy-(9Z)-octadecenoate (11H-12,13E-LA) and the already known 13-hydroxy-9,10-*trans*-epoxy-(11E)-octadecenoate (13H-9,10E-LA) were increased. These lipids were also capable of sensitizing primary afferent DRG neurons and induction of thermal hypersensitivity (Ramsden et al., 2017). Interestingly, in the amygdala, the concentrations of 13H-9,10E-LA were reduced in chronic inflammatory pain (Jensen et al., 2018). This metabolite is suggested to be synthesized by conversion of HODEs through CYP450, whereas the synthesis pathway of 11H-12,13E-LA and other metabolites is still unclear (Ramsden et al., 2017). They may be released by phospholipases or may be metabolized by peroxidation followed by hydroperoxide isomerization of LA (Ramsden et al., 2017). The concentrations of 11H-12,13E-LA are positively correlated with the occurrence of daily headaches in patients. Thus, lowering dietary intake of LA caused a reduction of 11H-12,13E-LA concentrations in plasma (Ramsden et al., 2017).

Not only in plasma was a correlation of the LA metabolites and pain observed. In psoriatic lesions the LA metabolite 9-keto-12,13-*trans*-epoxy-10E-octadecenoate (9K-12,13E-LA) was found to be increased and induced itch relating behavior (Ramsden et al., 2017).

Likewise, other lipids seem to influence chronic and persistent pain. The enzyme phospholipase A_2_ (PLA_2_), responsible for liberating AA of the phospholipid membrane, hydrolyzes the ester bond of glycerophospholipids to release polyunsaturated fatty acids and lysophopholipids like lysophosphatidylcholine (LPC) (Murakami and Kudo, 2002; Andersson et al., 2007]. LPC increases the temperature activation threshold of TRPM8 and thus increase cold sensitivity (Andersson et al., 2007; Gentry et al., 2010; Dennis and Norris, 2015). Decrease of LPC by inhibition of cytosolic calcium independent PLA_2_ (iPLA_2_) causes a reduction of calcium influx. When iPLA_2_ synthesizes LPC, the LPC further leads to prolonged TRPM8 channel opening, suggesting LPC as potent TRPM8 activator (Vanden Abeele et al., 2006) (**Table 1C**).

**Table 1C:** Other recently described lipids in inflammation and pain.

Animals/tissue/cell type	Effect	Signaling	Experiment	Experimental details	Target	Refs.
**NPD1 (precursor: DHA)**						
C57BL/6 mice, GPR37-KO mice and Cx3cr1-GFP mice, spinal cord, brain and skin tissue, DRGs, peripheral macrophages, CD1 mice	Antiinflammatory, antinociceptive	Reduction of IL-1β, CCL2 expression, phosphorylation of p38 and ERK, Increase of phagocytosis, expression of IL-10 and TGF-β	*In vivo, in vitro*	zymosan inflammatory pain model, intraplanar drug injection, *in situ* hybridization, LacZ staining, IHC, ELISA for IL-1β, TGF-β and IL-10, qPCR, Western Blot analysis, transfection, dot blot assay for lipid-binding protein, Ca-imaging, flow cytometry, phagocytosis assay by epifluorescence microscopy, licking or flinching time measurement, mechanical hypersensitivity *via* von Frey test, Randall-Selitto, behavioral studies, rotarod test, CCI model, with local peri-surgical pre-treatment of NPD1/PD1, autotomy/axotomy and chamber preference measurements with clonidine, long-term potentiation (LTP) measurements, mechanical allodynia studies, sEPSCs measurements	GPR37	(Xu et al., 2013b; Bang et al., 2018)
**S1P**						
Sprague-Dawley rats, Slprl KO and KD mice, human multiple myeloma cells	Proinflammatory	LPA receptor expression, TNFα and IL-1β release, decrease anti-inflammatory cytokines, p38 signaling, LPC activation	*In vivo*	intrathecal catheters or osmotic minipump for administration of compounds like fingolimod, D24 or D25, bortezomib injection intra peritoneal, S1pr1 silencing with 27mer dicer-substrate silencer RNA (DsiRNA), mechanical allodynia and hyperalgesia tests *via* von Frey and the Randall and Sellitto paw pressure test, mass spectrometry, Western Blot analysis, S1PR1 knockdown PCR, immunofluorescence, cytokine assay *via* multiplex cytokine kit, *in vitro* whole-cell recordings, tumor cell-killing assay *via* MTT assay		(Blaho and Hla, 2011; Stockstill et al., 2018)
**LPC (precursor: phospholipids)**						
CHO cells expressing mouse TRPM8, DRG cultures, Wistar rats, WT and TRPA1-KO mice, HEK-293 cells	Pronociceptive	Cold sensitivity	*In vivo, in vitro*	Ca-imaging, electrophysiology, intracellular calcium assay, subcutaneous injection in paw of icilin, menthol, LPC, BEL, saline, α,β-methylene ATP, Allyl isothiocyanate (AITC), cinnamaldehyde, cold plate and acetone evaporation measurement, heat sensitivity by hot-plate measurements, mechanical sensitivity transfection, iPLA2 activity assay, electrophysiology, fluorescence measurements of cytosolic calcium concentrations	TPM8, iPLA2	(Vanden Abeele et al., 2006; Andersson et al., 2007; Gentry et al., 2010; Dennis and Norris, 2015)
**LPA (precursor: lysophospholipids)**						
Neurons	Pronociceptive at early stage neuro-pathic pain	Increasing histamine release, demyelination, PKC and RhoA-ROCK-JNK signaling, Pro-liferation, calcium signaling, ATP release, ERK signaling, early stage neuropathic pain		See references (reviews)	LPAR_1_	(Blaho and Hla, 2011; Ueda, 2011)

Ca, calcium; CCL, chemokine ligand; CXCR1, C-X-C chemokine receptor type 1; DRGs, dorsal root ganglia; ELISA, enzyme-linked immunosorbent assay; ERK, extracellular-signal regulated kinase; IHC, immunohistochemistry; IL, interleukin; iPLA2, calcium-independent phospholipase 2; KD, knockdown; LPA, lysophosphatidic acid; LPC, lysophosphatidylcholine; LTP, long-term potentiation; MTT, 3(4,5-dimethylthiazol-2-yl)-2,5-diphenyltetrazolium bromide; sEPSCs, spontaneous excitatory post synaptic currents; TGF, transforming growth factor; NPD1, neuroprotectin D1; S1P, sphingosine-1 phosphate; S1PR1, S1P receptor 1; DHA, docosahexaenoic acid; TNF, tumor necrosis factor; LPAR, lysophosphatidic acid receptor; TRPM8, transient receptor potential melastatin ion channel 8; ROCK, Rho-associated, coiled-coil-containing protein kinase; PKC, protein kinase C; GPR, G-protein coupled receptor.

Upon intense stimulation of spinal cord neurons LPC is generated followed by a conversion through the extracellular lysophospholipase D autotaxin to lysophosphatidic acid (LPA) (Ueda, 2011). The roles of LPA in neuropathic pain are reviewed elsewhere in more detail (Ueda, 2011). In general, LPA leads to activation of peripheral nociceptor endings by increasing pain transmission, PKCγ signaling and leading to the release of histamine (Ueda, 2011). Furthermore, LPA activates the LPA_1_ receptor and by this RhoA-rho-associated, coiled-coil-containing protein kinase 1 (ROCK)-JNK signaling resulting in a negative regulation of myelin protein gene expression, which may cause demyelination (Ueda, 2011). Studies indicate that LPA induces calcium signaling in microglia, as well as proliferation, adenosine triphosphate (ATP) release and ERK signaling during the early stage of neuropathic pain development (Blaho and Hla, 2011; Ueda, 2011).

The expression of the LPA1 receptor is altered through signaling of sphingosine-1-phosphate (S1P), which is altered in chemotherapy-induced peripheral neuropathy (CIPN) and associated pain (Blaho and Hla, 2011; Stockstill et al., 2018). S1P signaling induces TNFα and IL-1β release, decrease of anti-inflammatory cytokines and activation of p38 signaling (Blaho and Hla, 2011; Stockstill et al., 2018). Apart from that, *ex vivo* S1P mediated LPC activation (Blaho and Hla, 2011).

Nerve-injury induced neuropathic pain and ongoing pain can be reduced by application of neuroprotectin D1 (NPD1) (Xu et al., 2013b). It can be synthesized from LA, which is converted through desaturases into eicosapentaenoic, into docosahexaenoic acid and then to NPD1 (Coste TC et al., 2003; Xu et al., 2013b; Dennis and Norris, 2015; Bang et al., 2018). NPD1 induces the inhibition of inflammation and inflammatory pain, by reducing IL-1β, C-C motif chemokine ligand 2 (CCL2) expression and phosphorylation of p38 and ERK (Xu et al., 2013b; Bang et al., 2018). This effect was a G_i_-mediated mechanism by the GPCR GPR37, which is mainly expressed on macrophages (Bang et al., 2018). Thereby NPD1 increases phagocytosis of macrophages, as well as expression of IL-10 and TGF-β in acute inflammation (Bang et al., 2018) (**Table 1C**). Whether the infiltration of inflammatory macrophages into DRG neurons is prevented through NPD1–GPR37 binding or by action of NPD1 itself is still unclear. However, treatment with NPD1 is effective in reducing neuropathic pain *in vivo*. It also shows no anti-nociceptive tolerance as opioids does and is capable of preventing diabetes-induced mechanical hypersensitivity (Xu et al., 2015). Thus, it seems to be a promising agent against neuropathic pain, which should be studied in more detail.

## Pharmacological Implications of Lipid Signaling in Chronic and/or Neuropathic Pain

The above-mentioned involvement of signaling lipids in different aspects of peripheral or central sensitization and their essential role in the pathophysiology of chronic and neuropathic pain raises the question, whether targeting synthesis, metabolism, or downstream signaling of these lipids may be a novel pharmacological strategy for the treatment of persistent pain states in patients. In this section, we therefore analyze and critically discuss the role of crucial proteins involved in lipid bioactivation and oxygenation, such as phospholipases and lipid oxygenases, lipid metabolism, such as soluble epoxide hydrolase, and lipid receptors, such as G2A and FFAR as targets for the treatment of chronic or neuropathic pain states.

The strongest evidence for the crucial involvement of lipid signaling in pain pathophysiology are prostaglandins, synthesized by cyclooxygenases COX-1 and COX-2. Both are targeted by non-steroidal anti-inflammatory drugs (NSAIDs), such as ibuprofen and diclofenac, which are widely used for the treatment of inflammatory pain (Day and Garry, 2013).

The significance of prostaglandins in inflammatory pain suggests an equally important role for other lipids in the pathophysiological processes of different pain states and that their regulating enzymes may represent targets for the development of novel analgesics, as shown by recent studies.

### Phospholipases

The crucial enzymes for bioactivation of signaling lipids and for releasing them from complex membrane forming lipids are phospholipases. In this group of enzymes, cPLA2, iPLA2, and the extracellular phospholipase D2 autotaxin seem to be particularly important for the release of fatty acids and lysophospholipids (Astudillo et al., 2018; Law et al., 2019).

Both, cPLA2 and iPLA2, are highly expressed in peripheral sensory neurons and central nervous neurons. cPLA2 cleaves fatty acids, such as linoleic acid and arachidonic acid by hydrolyzing the sn_2_ ester bond in phospholipids leading to bioactivation and liberation of these fatty acids (Farooqui et al., 2006; Sun et al., 2014). The activation of cPLA2 in peripheral sensory neurons is caused by increases in intracellular calcium concentrations through ion channels or release of intracellular calcium stores and by subsequent activation of Ca^2+^/calmodulin-dependent protein kinase II (CaMKII). These synergistic mechanisms lead to enhanced activity of cPLA2 which has been connected with increased neuronal activity and nerve-injury-induced neuropathic pain in preclinical studies (Tsuda et al., 2007; Hasegawa et al., 2009).

**Figure 2:**
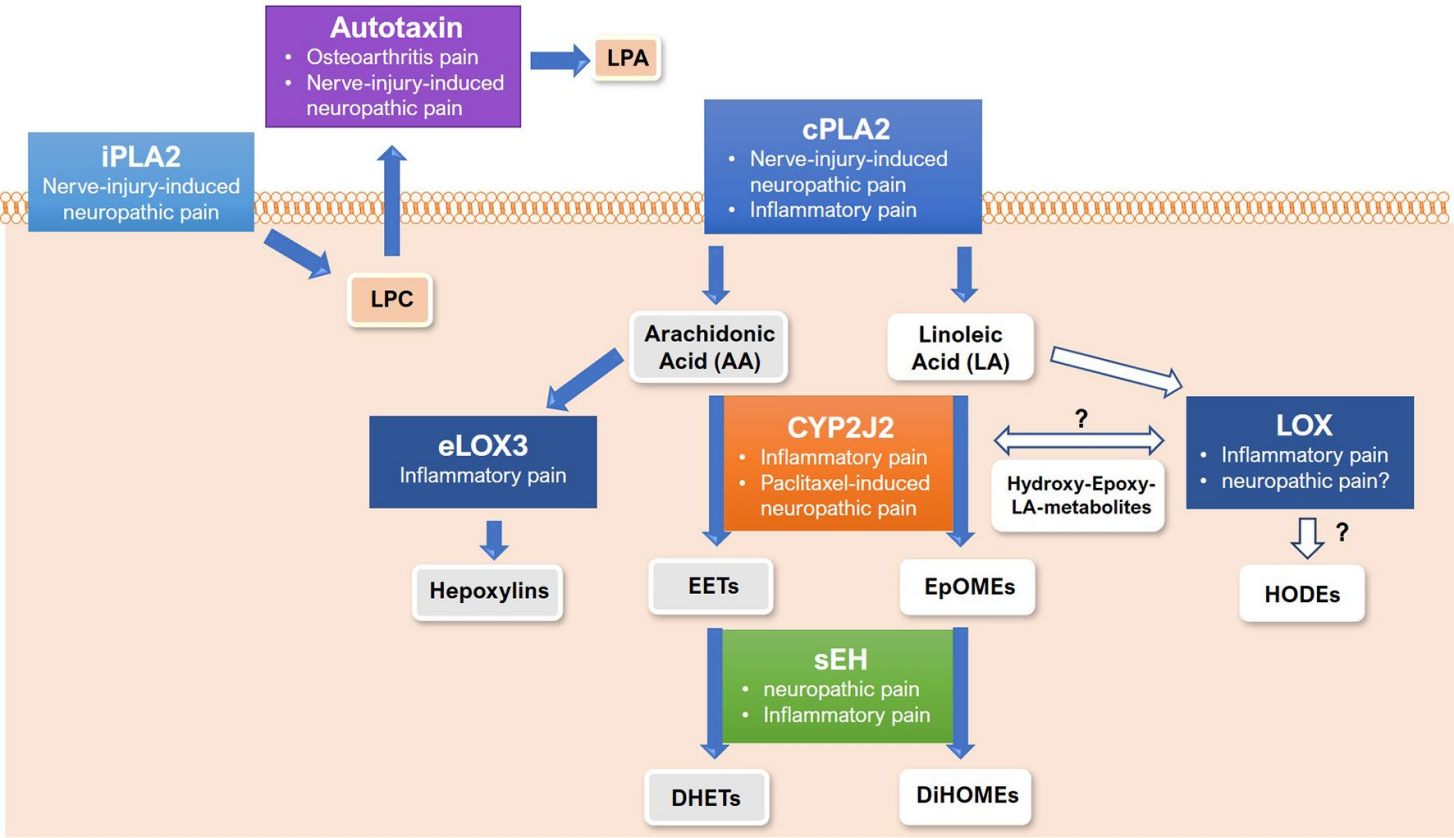
Proteins of lipid release, synthesis and metabolism in the pathology of persistent and/or neuropathic pain states. Shown are the proteins involved in release of lysophospholipids (in beige background), synthesis and metabolism of linoleic acid metabolites (in white background) or arachidonic acid metabolites (in grey background) and the respective pain state that each protein is connected with. White arrows indicate unknown synthesis or metabolism pathway. PLA, phospholipase; LA, linoleic acid; AA, arachidonic acid; LPC, lysophosphatidylcholine; CYP, cytochrome-P_450_-epoxygenase; LOX, lipoxygnease; sEH, soluble epoxide hydrolase; EET, epoxyeicosatrienoic acid; EpOME, epoxyoctadecadienoic acid; HODE, hydroxyotadecadienoic acid; DHET, dihydroxy-eicosatrienoic acid; DiHOME, dihydroxy-octadecenoic acid.

In contrast, iPLA2 activation does not depend on intracellular calcium changes. Other than cPLA2, iPLA2 cleaves the head group of complex phospholipids, resulting in release of lysophospholipids (Ramanadham et al., 2015). During nerve-injury-induced neuropathic pain, spinal cPLA2 and iPLA2-activity are increased. Thus, intrathecal injection of both the dual PLA2 inhibitor Arachidonyl trifluoromethyl ketone (AACOCF_3_) and the selective iPLA2 inhibitor bromoenol lactone (BEL) caused a reduction of lipid release in the spinal cord and neuropathic pain *in vivo* (Ma et al., 2010). Moreover, administration of the dual PLA2-inhibitor AACOCF_3_ reduced thermal hyperalgesia and formalin-induced flinching *in vivo* (Lucas et al., 2005) (**Table 2**, **Figure 2**) suggesting an involvement of PLAs in the pathophysiological processes leading to inflammatory and/or neuropathic pain.

**Table 2:** The role of proteins involved in lipid release, synthesis and metabolism, as well as lipid GPCRs in various pathophysiological mechanism of persistent and neuropathic pain.

Enzyme/receptor	Effect	Pathophysiological relevance	Effect of inhibitor or knock-out	Pain state investigated (model)	Refs.
cPLA2	Releases fatty acids (LA, AA) from membranes (sn_2_-position)	Increased in the PNS and CNS during inflammatory and neuropathic pain	Intrathecal injection of AACOCF_3_ caused reduction of inflammatory and neuropathic pain *in vivo*	Inflammatory pain (carrageenan, formalin), neuropathic pain (nerve injury)	(Lucas et al., 2005; Ma et al., 2010)
iPLA2	Releases fatty acids (LPC) from membranes	Increased in the CNS during neuropathic pain	Intrathecal injection of AACOCF_3_ and BEL caused reduction of neuropathic pain *in vivo*	neuropathic pain (nerve injury)	(Ma et al., 2010)
Autotaxin (PLD)	Converts LPCs to LPAs extracellularly	Increased in the plasma and synovial fluid of OA patients	Test compound reduced joint pain and fracture pain *in vivo*. Autotaxin-knock-out mice have reduced nerve injury- induced neuropathic pain.	MIA model for osteoarthritis and osteotomy model; partial sciatic nerve injury- induced neuropathic pain	(Inoue et al., 2008; Thirunavukkarasu et al., 2017)
CYP2J2	Oxidizes fatty acids (LA, AA)	Increased in the PNS during inflammatory and paclitaxel-induced neuropathic pain	Ketoconazole reduced CFA-induced inflammatory pain and Telmisartan reduced paclitaxel-induced mechanical hypersensitivity *in vivo*	CFA model for inflammatory pain, paclitaxel model for chemotherapy-induced neuropathic pain	(Ruparel et al., 2012a; Sisignano et al., 2016)
LOX	Oxidizes fatty acids (LA, AA)	Spinal eLOX3 increased during inflammatory pain. Microglial 15-LOX-1 increased during LPS-induced inflammatory pain	Intraplantar injection of NDGA caused reduction of heat hyperalgesia; systemic treatment with NDGA caused reduction of mechanical hypersensitivity	Dissolved compounds isolated from heated skin superfusates were injected intraplantar (model for inflammatory pain); carrageenan-induced inflammatory pain	(Buczynski et al., 2010; Patwardhan et al., 2010; Gregus et al., 2013; Gregus et al., 2018)
sEH	Hydrolyzes epoxylipids (EpOMEs, EETs)	Concentrations of anti-inflammatory epoxylipids are systemically increased by sEH inhibition	Systemic treatment of sEH causes a reduction of inflammatory pain and diabetes-induced neuropathic pain	LPS, carrageenan, zymosan and CFA-induced inflammatory pain, Streptozotocin (STZ)-induced neuropathic pain	(Inceoglu et al., 2006; Schmelzer et al., 2006; Inceoglu et al., 2008; Inceoglu et al., 2012; Zimmer et al., 2018)
G2A (GPR132)	Activated by 9-HODE and other lipids	Increased 9-HODE concentrations observed in neuronal tissue during oxaliplatin-induced neuropathic pain	G2A-deficient mice have less oxaliplatin-induced neuropathic pain and less zymosan-induced thermal hyperalgesia	Oxaliplatin-induced neuropathic pain, zymosan-induced peripheral inflammation and inflammatory pain	(Hohmann et al., 2017; Kern et al., 2018)
FFAR1 (GPR40)	Activated by medium- and long-chain fatty acids	Increased expression in DRG neurons during inflammatory and neuropathic pain	Selective agonists MEDICA16 or GW9508 cause a reduction of inflammatory and nerve-injury-induced neuropathic pain	CFA- and carrageenan model for inflammatory pain, formalin model for acute/inflammatory pain, SNL model for neuropathic pain	(Nakamoto et al., 2012; Karki et al., 2015)
LPAR1/3	Activated by lysophosphatidic acids of various chain lengths	LPAR1 activation causes increased neuronal activity, and Schwan cell dependent demyelination	LPAR1-deficient mice have reduced inflammatory and neuropathic pain, both LPAR1- and LPAR3-deficient mice have reduced paclitaxel-induced neuropathic pain	Partial sciatic nerve injury model for neuropathic pain (LPAR1); carrageenan-model for orofacial inflammatory pain (LPAR1) Paclitaxel model for chemotherapy-induced neuropathic pain LPAR1/3);	(Inoue et al., 2004; Uchida et al., 2014; Srikanth et al., 2018)
S1P1R	Activated by sphingosine-1-phosphate (S1P)	S1P1-activation causes activation of spinal astrocytes after bortezomib-treatment and after nerve injury	Mice deficient of astrocytes expressing S1P1R have reduce bortezomib-induced neuropathic pain; pharmacological blockade of the S1P1R causes reduction of bortezomib-induced and CCI-induced neuropathic pain	Bortezomib-induced neuropathic pain CCI models of nerve injury induced neuropathic pain	(Stockstill et al., 2018; Chen et al., 2019)
BLT1/2	Activated by leukotriene B_4_ (LTB_4_)	BLT1 activation causes TRPV1 sensitization, BLT2 activation reduces TRPV1 sensitization in sensory neurons	Mice treated with a BLT2 agonist show reduced mechanical and thermal hypersensitivity during inflammatory pain	Zymosan model for inflammatory pain	(Zinn et al., 2017)
GRP37	Activated by Neuroprotectin D1 (NPD1)	Causes increases of [Ca^2+^]_i_ in macrophages and promotes phagocytosis and resolution of inflammation	GPR37-knock-out mice have reduced inflammatory pain (heat hyperalgesia and mechanical allodynia)	Zymosan model for inflammatory pain	(Bang et al., 2018)

AA, arachidonic acid; BEL, bromoenol lactone; BLT, B-Leukotriene receptor; CCI, chronic constriction injury; CFA, complete Freund’s adjuvant; CNS, central nervous system; CYP, cytochrome- P_450-_epoxygenase; DRG, dorsal root ganglion; EET, epoxyeicosatrienoic acid; EpOME, epoxyoctadecadienoic acid; FFAR, free fatty acid receptor; G2A, G2-accumulating GPCR; HODE, hydroxyotadecadienoic acid; LA, linoleic acid; LPAR, lysophosphatidic acid receptor; LPC, lysophosphatidylcholine; LPS, lipopolysaccharide; LOX, lipoxygenease; LTB_4_, leukotriene B_4_, MIA, monoiodoacetate; NDGA, nordihydroguaiaretic acid; NPD, neuroprotectin D1; PNS, peripheral nervous system; PLA/PLD, phospholipases; Refs, references; sEH, soluble epoxide hydrolase; SNL, spinal nerve ligation; S1P, sphingosine-1-phosphate; S1PR1, sphingosine-1-phosphate receptor 1; STZ, Streptozotocin; TRPV1, transient receptor potential vanilloid 1 channel.

In contrast to the intracellular acting PLAs, autotaxin is an extracellular phospholipase D that processes released lysophospholipids, such as lysophosphaditylcholine, by removing the choline head group. This results in the bioactivation of free lysophosphatidic acids (Albers and Ovaa, 2012). In plasma and synovial fluid of patients suffering from knee osteoarthritis and associated pain, autotaxin is markedly increased and seems to correlate with severity of osteoarthritis in patients (Mabey et al., 2015). Recently, potent and orally available inhibitors of autotaxin have been developed (Jones et al., 2016). Administration of one test compound caused a decrease of joint pain in the mono-sodium iodoacetate (MIA) model of osteoarthritis as well as a reduction of bone fracture pain in the osteotomy model in rodents (Thirunavukkarasu et al., 2017) (**Figure 2**, **Table 2**). Moreover, in the pathophysiological process of nerve-injury-induced neuropathic pain, LPAs seem to contribute to Schwann cell degradation and demyelination (Inoue et al., 2008). However, the role of autotaxin inhibitors in this context has not yet been investigated.

These observations imply a role for phospholipases in pain pathophysiology. However, the central role of these enzymes in the bioactivation of signaling lipids in many different contexts hamper specific targeting of these enzymes in chronic and/or neuropathic pain states respectively. For example, cPLA2 seems to play a role in a variety of diseases which involve transient or chronic inflammation, such as brain injury, pulmonary fibrosis and Alzheimer’s disease (Dennis et al., 2011). However, the role of cPLA2 in these pathophysiological contexts seems to be contrasting, as it’s metabolites may have beneficial effects for example in cardiac fibrosis, but may also promote disease progression and severity, as for example in allergic reactions (Aloulou et al., 2012). Similar contrasting effects are known for iPLA2 in many different disease states (Ramanadham et al., 2015).

These contrasting roles and the essential requirement of phospholipases in many physiological and pathophysiological conditions, speak against systemic targeting of phospholipases during pain treatment, as many unwanted effects are likely to occur. In this regard, it may be more useful to apply phospholipase inhibitors more locally and transiently to inhibit phospholipases only in specific cellular subpopulations and only at the time of their aberrant activity. However, until now, there is no pharmacological inhibitor of phospholipases available for clinical use. In general, more research is required to understand the specific contribution of phospholipases to persistent pain states, as well as their temporal activity in pain pathophysiology that may allow targeting these enzymes for the treatment of chronic or neuropathic pain in patients.

### Lipid Oxygenases — CYP and LOX

The liberation of fatty acids from membrane lipids by cPLA2 makes them available as substrates for lipid oxygenases. There are three main classes of lipid oxygenases: 1. the above-mentioned cyclooxygenases (COX), 2. lipoxygenases (LOX) and 3. cytochrom-P_450_-epoxygenases (CYP). Because the role of COX and its prostanoid metabolites in persistent pain states have been reviewed previously (Eisenach et al., 2010; Chen et al., 2013), we focus on recent observations involving LOX and CYP enzymes in inflammatory or neuropathic pain states.

Lipoxygenases catalyse their reaction with iron and CYP-epoxygenases contain a full cytochrome as cofactor. Lipoxygenases usually attach a hydroperoxide group onto one of the substrates’ double bonds that is subsequently reduced to a hydroxide group. CYP-epoxygenases attach epoxide groups or terminal hydroxide groups to their substrate fatty acids. These enzymes can oxidize a variety of different substrates, but the most abundant are the ω-6 fatty acids linoleic acid (giving rise to oxidized linoleic acid metabolites) and arachidonic acid (giving rise to oxidized eicosanoids, **Figure 2**) (Haeggstrom and Funk, 2011; Xu et al., 2013a; Spector and Kim, 2015).

Several lines of evidence suggest a crucial role for CYP-epoxygenases of the subfamily 2J in the pathogenesis of persistent or neuropathic pain.

The expression of the genes encoding for the CYP-epoxygenase isoforms 2J and 3A was elevated in dental pulp samples of patients with inflammatory dental pain as well as in trigeminal neurons of rats during complete Freund’s adjuvant (CFA)-induced inflammatory pain (Ruparel et al., 2012b; Ruparel et al., 2013). In this context, treatment of rodents with the unspecific CYP-epoxygenase inhibitor ketoconazole caused a reduction of pain hypersensitivity during CFA-induced inflammation (Ruparel et al., 2012a).

CYP-epoxygenases are essential proteins for the biotransformation of xenobiotic substances. Therefore, exogenous and potentially toxic substances may trigger increased activity of CYP-epoxygenases (Murray, 2016). Indeed, it was shown that paclitaxel, a cytostatic that is widely used for the treatment of breast cancer and that causes neuropathic pain in patients, can enter peripheral sensory neurons easily (Park et al., 2013). In a preclinical study it was observed that paclitaxel causes increased expression of the gene encoding for the CYP-epoxygenase isoform 2J6 (CYP2J6, in human: CYP2J2) in murine sensory neurons. Administration of the CYP2J2 inhibitor Telmisartan was sufficient to reduce neuronal synthesis of CYP2J2 derived oxidized lipids and to reduce mechanical hypersensitivity of mice during paclitaxel-induced neuropathic pain in a dose-dependent and preventive manner (Sisignano et al., 2016) (**Figure 2**, **Table 2**).

Apart from CYP-epoxygenases, available free linoleic and arachidonic acid can also be converted by lipoxygenases (LOX).

As stated above, the hydroxylated linoleic acid metabolites 9- and 13-HODE show pronociceptive effects in preclinical inflammatory and neuropathic pain models. Unlike other lipids discussed in the first chapter, the synthesizing enzyme for HODEs, particularly for 9-HODE, remains unclear. Likewise, for novel linoleic acid-derived hydroxy-epoxy or keto epoxy-mediators that have been described to contribute to pain and itch (Ramsden et al., 2017), the synthesis pathway and the involvement of specific lipoxygenases and/or CYP-epoxygenases is unclear. However, the presence of non-terminal hydroxide groups in these lipids implies a role for LOX enzymes in their synthesis.

In the state of inflammatory pain, the expression of the gene encoding for eLOX3 was found increased in the spinal cord. Additionally, microglial expression of the gene encoding for 15-LOX-1 was elevated after LPS-induced induction of spinal toll-like receptor 4 (TLR4) in rodents, pointing towards induction of specific spinal lipoxygenase isoforms during inflammatory pain (Gregus et al., 2013; Gregus et al., 2018). Indeed, systemic treatment with the unspecific LOX inhibitor nordihydroguaiaretic acid (NDGA) caused a reduction of spinal LOX-derived lipids and led to a decrease of mechanical hypersensitivity in rats during carrageenan-induced inflammatory pain (Buczynski et al., 2010). In another study, dissolved compounds isolated from heated skin superfusates were injected intraplantar to induce a peripheral inflammation and inflammatory pain. In this model, peripheral intraplantar injection of the LOX inhibitor NDGA in the inflamed paw of rodents caused a reduction of inflammatory heat hyperalgesia *in vivo* (Patwardhan et al., 2010) (**Figure 2**, **Table 2**).

In summary, these results are promising and indicate a crucial role for CYP2J2 in paclitaxel-induced neuropathic pain and for both CYP2J2 and specific lipoxygenase isoforms in inflammatory pain. Telmisartan can be easily repurposed as CYP2J2 inhibitor due to long clinical experience with this drug and its good tolerability in patients (Battershill and Scott, 2006), whereas the unselective antifungal CYP-inhibitor ketoconazole is not suitable for systemic use due to high cross-reactivity, and it may cause hepatotoxicity upon systemic administration (Gupta and Lyons, 2015).

For the above-mentioned lipoxygenases eLOX3 and 15-LOX-1 specific and clinically available inhibitors are lacking. The unspecific inhibitor NDGA that was used in rodent models is not suitable for clinical use in patients and may cause unwanted side effects. Moreover, it may inhibit isoforms of lipoxygenases that are required for normal lipid homeostasis under physiological conditions. Therefore, more research is required on identification and clinical development of safe and specific inhibitors of lipoxygenase isoforms that are dysregulated in persistent pain states. These substances may be used as novel analgesics for the treatment of persistent and neuropathic pain in patients.

### Soluble Epoxide Hydrolase

Soluble epoxide hydrolase (sEH) is the enzyme that converts epoxylipids, such as EpOMEs and EETs to diol lipids by hydrolysis of the epoxide group (Yu et al., 2000). This enzyme has a unique dual functioning role, as it contains both a hydrolase domain, that is responsible for lipid hydrolysis, and a lipid phosphatase domain which may be involved in the metabolism of lysophosphatidic acids (LPAs) (Newman et al., 2003; Morisseau et al., 2012). However, the role of the phosphatase group, especially in pathophysiological states, is unknown, and the available inhibitors of sEH mainly target the hydrolase domain (Shen and Hammock, 2012).

Inhibiting sEH leads to an increase in cellular and systemic epoxylipid concentration and a decrease in the concentrations of diol-metabolites. Because epoxy-metabolites of arachidonic acid (EETs) have previously been identified as potent vasodilators, and sEH-inhibition leads to their systemic accumulation, sEH inhibitors were considered as promising novel pharmacological therapies for the treatment of cardiovascular diseases (Imig and Hammock, 2009). However, later studies demonstrated the involvement of epoxylipids, diol-metabolites, and sEH as their regulatory enzyme in the pathogenesis of persistent and neuropathic pain.

It was shown that the inhibition of sEH can reduce the levels of the proinflammatory and proalgesic mediator prostaglandin E_2_. sEH may also reduce the activity of spinal COX-2 during inflammatory pain, which points towards a cross-regulation of CYP and COX pathways. Moreover, treatment of rodents with sEH inhibitors caused a reduction of inflammatory pain hypersensitivity *in vivo* (Inceoglu et al., 2006; Schmelzer et al., 2006; Inceoglu et al., 2008) (**Table 2**, **Figure 2**).

Interestingly, the analgesic efficacy of sEH inhibitors seems to be not only restricted to inflammatory pain. In a rat model of streptozotozin (STZ)-induced diabetic neuropathy, the administration of three different sEH-inhibitors caused a reduction of mechanical hypersensitivity *in vivo*. This effect was similarly strong as the analgesic effect of the calcium channel blocker gabapentin (Inceoglu et al., 2012).

The antihyperalgesic effects of sEH inhibitors in preclinical studies are impressive and suggest sEH as promising target for the development of novel analgesics but there are still many open questions and limitations of these compounds for a clinical use in patients. The mechanistic basis of their actions has long been attributed to systemic increase of epoxylipid concentrations and their anti-inflammatory actions (Thomson et al., 2012).

Moreover, the diol metabolites of sEH were previously considered to be biologically less active than their parent epoxilipids (Spector and Norris, 2007). However, this image is challenged by recent studies and there is evidence for the involvement of diol-lipids in several pathophysiological mechanisms. In one study, the linoleic acid-derived diol metabolite of sEH, 12,13-DiHOME was identified as TRPV1-sensitizing mediator that is produced in neuronal tissue during zymosan- or CFA-induced inflammatory pain. Treatment of mice with the sEH inhibitor TPPU causes a reduction of 12,13-DiHOMEs synthesis in neuronal tissue and reduces both CFA- and zymosan-induced thermal hyperalgesia *in vivo* (Zimmer et al., 2018) (**Figure 2**, **Table 2**). Similarly, the sEH metabolite 19,20-DHDP has recently been identified as crucial mediator of diabetic retinopathy and treatment of mice with an sEH inhibitor caused a reduction of 19,20- Dihydroxydocosapentaenoic acid (19,20-DHDP) concentrations in the retina and a reduction of diabetic retinopathy *in vivo* (Hu et al., 2017).

These recent observations challenge the idea, that diol-metabolites are less active than their parent epoxylipids. Moreover, these results indicate that the effects of sEH-inhibitors may be caused not just by increasing epoxylipid concentrations, but by a simultaneous decrease of diol-lipid concentrations.

However, it is still unknown which physiological and pathophysiological role the phosphatase domain of sEH has. It has been proposed that the phosphatase domain may be relevant for the metabolization of lysophosphatidic acids (LPAs) (Morisseau et al., 2012). This indicates that sEH may be a crucial regulator at the interface of both lysophospholipid and fatty acid metabolism, yet more research is required to identify the role of this enzyme and in particular, the regulatory functions of the phosphatase domain in physiological and pathophysiological processes.

Additionally, it remains to be investigated which potential side effects sEH inhibitors may cause. For example, the sEH inhibitor 12-(3-adamantan-1-yl-ureido)-dodecanoic acid (AUDA), that was used in early *in vivo* studies, was found to directly activate the peroxisome proliferator-activated receptor (PPAR)-α (Fang et al., 2005). This receptor may in part be responsible for some of its anti-inflammatory effects independent of lipid-mediated effects. However, newer soluble epoxide hydrolase inhibitors seem to be more specific (Lee et al., 2014). Indeed, selected sEH-inhibitors have already proceeded to clinical testing in inflammatory contexts (clinicaltrials.gov), but conclusive results concerning their effects in patients have not yet been published.

### Lipid GPCRs

**Figure 3:**
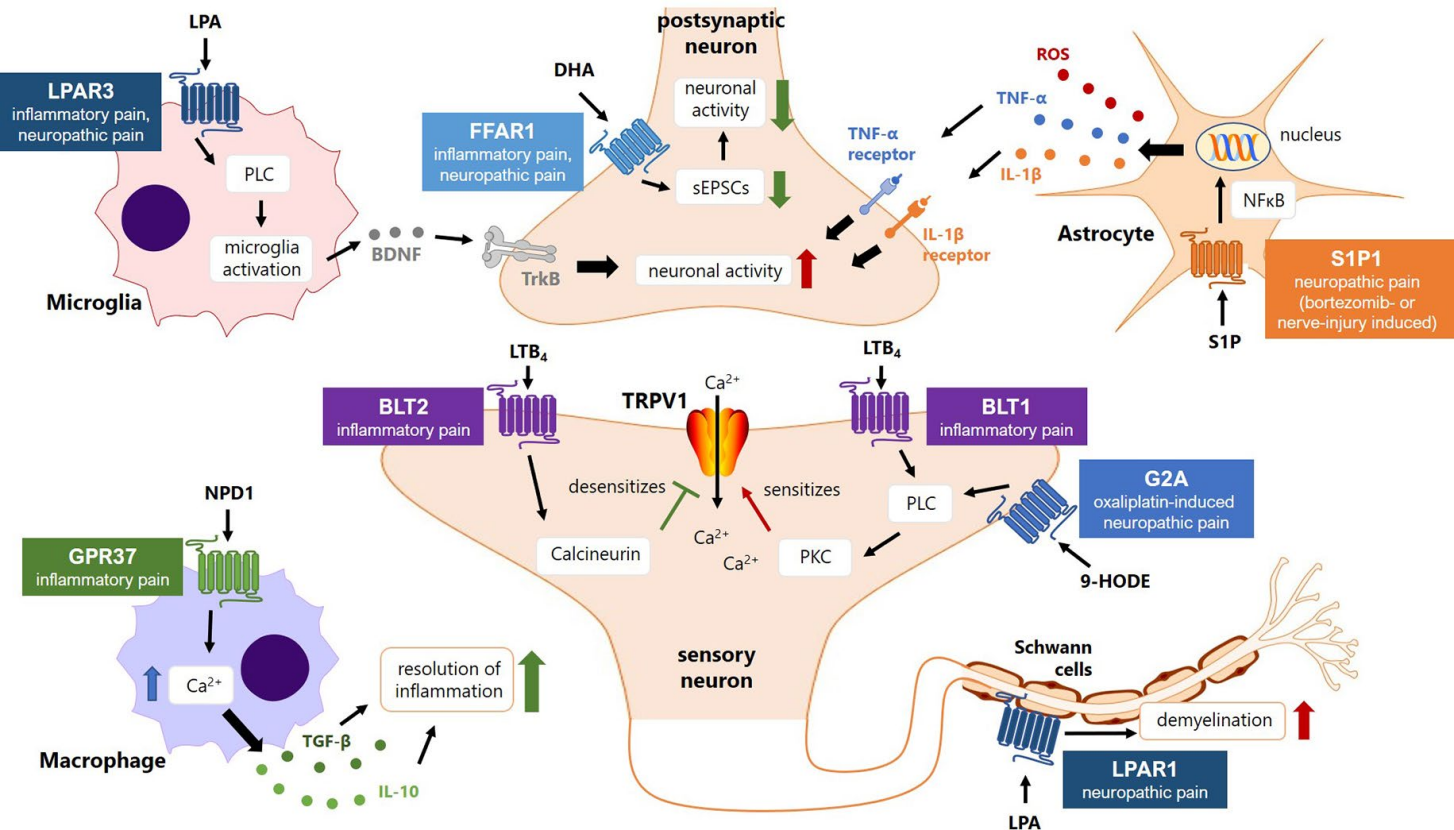
Lipid GPCRs in the pathology of persistent and/or neuropathic pain states. Shown are the six lipid GPCRs that are discussed in the manuscript with an exemplary lipid ligand and their respective intracellular signaling pathways, their cellular distributions and signaling functions in persistent pain states at the interface of the peripheral and central nervous system. LPA, lysophosphatidic acid; LPAR, lysophosphatidic acid receptor; PLC, phospholipase C; BDNF, brain-derived neurotrophic factor; DHA, docosahexaenoic acid; FFAR, free fatty acid receptor; GPR, G-protein coupled receptor; sEPSCs, spontaneous excitatory postsynaptic currents; TNF-α, tumor necrose factor-alpha; IL-1β, interleukin-1-beta; S1P, sphingosine-1-phosphate; S1P1R, sphingosine-1-phosphate receptor; NFκB, nuclear factor kappa-light-chain-enhancer of activated B-cells; HODE, hydroxyotadecadienoic acid; G2A, G2-accumulating GPCR; LTB4, leukotriene B_4_; BLT, B-leukotriene receptor; PKC, protein kinase C; TRPV1, transient receptor potential vanilloid 1 channel; NPD1, neuroprotectin D1; TGF, transforming growth factor-beta; IL-10, Interleukin-10.

G-protein coupled receptors (GPCRs) are still the most important target proteins in pharmacology and by now the high-resolution structures of more than 50 individual GPCRs have been solved (Alexander et al., 2017). Among these GPCRs are also specific receptors for signaling lipids which contribute to the transmission of cell-cell-signals in physiological and pathophysiological conditions. The pain relevant GPCRs comprise of prostanoid receptors, cannabinoid receptors, leukotriene receptors, as well as LPA receptor 1 and 3, S1P receptor 1, G2A, FFAR1 (GPR40) and GPR37 (Audet and Stevens, 2019). While the various functions of prostanoid and cannabinoid receptors have previously been reviewed (Manzanares et al., 2006; Narumiya, 2007), we focus on recently described lipid GPCRs that may be involved in pathogenesis of persistent and neuropathic pain and may represent targets for the development of novel analgesics (**Figure 3**).

#### Fatty Acid GPCRs — G2A and FFAR1

The PTX-sensitive G2A receptor (GPR132) was initially described to be accumulating in the G_2_/M phase of the cell cycle and generally seems to be induced by DNA damage and related cellular stress. Due to the accumulation of the receptor in the G_2_ phase it was named G2A(ccumulating) receptor (Weng et al., 1998). By means of sequence analysis and comparison to different classes of GPCRs, G2A was categorized within the group of proton-sensing GPCRs and seems to be activated by mild pH reduction (Murakami et al., 2004). However, G2A lacks crucial histidine residues, that all other proton-sensing GPCRs [GPR4, T-cell death associated gene 8 (TDAG8) and Ovarian cancer G-protein coupled receptor 1 (OGR1)] contain and is therefore only weakly activated by pH reduction (Radu et al., 2005). In contrast, heterologous expressed G2A can be activated by various oxidized eicosanoids and linoleic acid metabolites. The strongest activator seems to be the oxidized linoleic acid metabolite 9-HODE (Obinata et al., 2005).

G2A is expressed in immune cells, such as macrophages, neutrophils, and T-cells, but also in a subset of TRPV1- and isolectin B4 (IB4)-positive primary sensory neurons in the dorsal root ganglia (Kwan et al., 2007; Justus et al., 2013). In these neurons, G2A seems to be activated by excessive 9-HODE and seems to contribute to TRPV1 sensitization *via* G_q_-coupling and activation of PKC during oxaliplatin-induced neuropathic pain. This causes increased activity of sensory neurons, increased release of the proinflammatory neuropeptide CGRP and contributes to oxaliplatin-induced mechanical hypersensitivity *in vivo*. Indeed, the oxaliplatin-induced mechanical hypersensitivity is significantly reduced in G2A-deficient mice (Hohmann et al., 2017). In macrophages, G2A seems to be responsible for mechanisms involved in migration. Indeed, G2A-deficient mice that were peripherally injected with zymosan, showed reduced numbers of infiltrating macrophages and a decreased intensity of zymosan-induced thermal hyperalgesia compared with wild-type mice (Kern et al., 2018).

These observations point towards a central role of G2A in sensitization processes of peripheral sensory neurons either by direct activation of neuronal PKC and sensitization of TRPV1 or indirectly, by influencing immune cell migration in local inflammations (**Figure 3**, **Table 2**).

However, there are currently no selective inhibitors of G2A available which hamper characterization of the role of this receptor in pain pathogenesis and other pathophysiological states. A G2A inhibitor may be used as leads for clinical development. Moreover, the role of G2A under physiological conditions is not well understood and recent studies point towards involvement of this receptor in hematopoiesis (Lahvic et al., 2018).

In summary, although G2A may be a promising target for the development of novel analgesics, research on this receptor is currently at a very early stage and many open questions remain to be answered.

The free-fatty acid receptor 1 FFAR1 (GRP40) is activated by medium- and long-chain fatty acids, plays an important role in glucose homeostasis and insulin secretion and is a long known and well-studied target for the treatment of type 2 diabetes (Wagner et al., 2013).

However, FFAR1 also seems to be expressed in peripheral sensory neurons in the dorsal root ganglia, as well as in spinal cord neurons. Indeed, intrathecal injection of the selective FFAR1 agonists MEDICA16 or GW9508 caused a reduction of mechanical hypersensitivity in CFA-induced inflammatory pain and spinal nerve ligation (SNL)-induced neuropathic pain, as well as a reduction of thermal hyperalgesia in carrageenan-induced inflammatory pain *in vivo* (Karki et al., 2015). This confirms previous results, that intrathecal injection of GW9508 reduced formalin-induced pain behavior in mice possibly *via* increase of hypothalamic β-endorphin (Nakamoto et al., 2012).

Apart from a potential involvement of the endogenous opioid system, the mechanistic basis of the analgesic effects of FFAR1-agonists in various pain models is still unclear. It should be investigated whether these effects are indeed mediated by FFAR1 or caused by unspecific off-target effects by the FFAR1 agonists. In this regard, more research is required to identify the mechanistic involvement of FFAR1 in sensitization and increased activity of peripheral sensory neurons during inflammatory and neuropathic pain. The fact that FFAR1 agonists are already tested in clinical trials (clinicaltrials.gov) and may be available for diabetes patients in the near future, may facilitate repurposing of these compounds for the treatment of chronic or neuropathic pain (**Figure 3**, **Table 2**).

#### Lysophospholipid Receptors — LPR1 and 3 and S1P1R

By now, six lysophosphatidic acid receptors have been identified (LPAR1-6) with various roles in different tissues and pathophysiological contexts (Yung et al., 2014). The role of lysophosphatidic acid and its receptor LPAR1 in neuropathic pain has first been described in 2004 by Inoue et al. (Inoue et al., 2004). The authors showed that intrathecal injection of LPA directly causes mechanical hypersensitivity in rodents and that mice deficient of LPAR1 showed reduced mechanical hypersensitivity in the partial sciatic nerve injury model of neuropathic pain (Inoue et al., 2004). While LPAR1 is the main LPA receptor expressed in DRG neurons and its activation causes increased neuronal activity, it is also expressed in Schwann cells and seems to be involved in demyelination processes that occur at the onset of neuropathic pain (Ueda et al., 2013). Likewise, it has been observed that both, mice that are deficient of the LPAR1 receptor, as well as pharmacological inhibition of LPAR1 cause a reduction of mechanical hypersensitivity in a carrageenan-induced orofacial model of inflammatory pain (Srikanth et al., 2018). It was also described that LPAR1- or LPAR3-deficient mice show reduced paclitaxel-induced mechanical hypersensitivity (Uchida et al., 2014). Moreover, it was shown that the lipid LPA18:1, which is a ligand for the LPA receptors, also causes direct activation of TRPV1 in sensory neurons by interacting with a C-terminal region of the ion channel (Nieto-Posadas et al., 2011).

These observations in various pain models point towards a central role of the LPA receptor system in modulating neuronal activity, Schwann cell dependent demyelination and inflammatory mechanisms in persistent pain states. However, it remains pharmacologically challenging to selectively target LPAR1 or both LPAR1 and LPAR3 with one compound, without affecting the activity of the remaining four LPA receptors, all of which seem to have central physiological roles in various contexts (Yung et al., 2014). Global inhibition of all LPA receptors may therefore be connected with serious side effects and cannot be recommended. This selectivity problem currently restrains targeting the LPA receptor system in the context of inflammatory or neuropathic pain and requires more research and more selective targeting approaches to exploit the LPA receptor axis for the development of novel analgesics (**Figure 3**, **Table 2**).

The lipid sphingosine-1-phosphate (S1P) is one of the most important signaling lipids and regulates many physiological and pathophysiological processes in mammalian cells and tissues *via* its five G-protein coupled receptors (S1PR1-5) (Maceyka et al., 2012). A study identified a role for this lipid and specifically for the S1P1 receptor in chemotherapy-induced neuropathic pain caused by the proteasome inhibitor bortezomib. The authors of this study found that bortezomib-treatment increased spinal concentrations of S1P and other related lipids from its metabolic pathway. Moreover, bortezomib causes increased activity of spinal astrocytes *via* S1P1 receptor signaling. Treatment of mice with fingolimod or NIBR14, both of which are selective inhibitors of the S1P1 receptor, caused a reduction of bortezomib-induced mechanical hypersensitivity *in vivo* (Stockstill et al., 2018). Likewise, inhibition of the S1P1 receptor was found beneficial in chronic-constriction-injury (CCI)-induced neuropathic pain *in vivo* as published in a recent study. The authors show that nerve injury increases spinal S1P concentrations causing activation of S1P1 receptors in spinal astrocytes, leading to enhanced synaptic activity of sensory neurons. Inhibition of the astrocytic S1P receptor increases release of IL-10 which reduces the inflammatory response and leads to a decrease in synaptic activity of sensory neurons (Chen et al., 2019).

As discussed above, for the LPA receptors, there is currently no selective antagonist clinically available. In contrast, for the S1P1 receptor, fingolimod, a selective S1P1 receptor antagonist, is an already approved drug for the treatment of multiple sclerosis (Pelletier and Hafler, 2012). This could lead to repurposing of fingolimod for the treatment of bortezomib-induced- and nerve-injury induced neuropathic pain in patients (**Figure 3**, **Table 2**).

#### Leukotriene Receptors BLT1 and 2 and the Neuroprotectin Receptor GPR37

The signaling lipid leukotriene B_4_ that is involved in inflammatory processes and can bind two G-protein coupled receptors, called B-leukotriene receptors (BLT1 and BLT2) (Yokomizo, 2015). A recent study investigated the expression of GPCRs in peripheral sensory neurons of the dorsal root ganglia (DRG) and found that the two BLT receptors are among the 10 GPCRs with the strongest basal expression in DRGs (Zinn et al., 2017). Further investigation revealed that LTB_4_ shows a biphasic activity pattern in sensory neurons and binds BLT1 with high affinity and BLT2 with lower affinity. Moreover, the activation of the BLT1 receptor causes sensitization of the TRPV1 channel in these neurons. However, activation of the BLT2 receptor reduces sensitization of TRPV1 *via* activation of calcineurin. Treatment of mice with a BLT2 agonist causes a reduction of mechanical and thermal hypersensitivity in zymosan-induced inflammatory pain *in vivo* (Zinn et al., 2017). These results identify a self-regulating system of TRPV1 sensitization and neuronal activity by the two BLT receptors and indicate that inhibition of BLT1, activation of BLT2 or both mechanisms synergistically could be exploited for the development of novel analgesics in the context of increased TRPV1 sensitization during inflammatory pain.

The omega-3 lipid Neuroprotectin D1 (NPD1) that is synthesized from the precursor docosahexaenoic acid (DHA) has previously been shown to reduce neurogenic inflammation and mechanical hypersensitivity in nerve-injury models of neuropathic pain by reducing spinal activation of microglia and subsequent release of the proinflammatory mediators TNF-α and CCL-2 (Xu et al., 2013b). However, it remained unclear which receptor is mediating these effects of NPD1. Recently, GPR37 was identified as putative NPD1 receptor, and the lipid causes increases in intracellular calcium in GPR37-transfected cells. Moreover, the NPD1-GPR37-calcium-increase axis seems to regulate phagocytosis of zymosan particles as well as cytokine release by macrophages and seems to promote resolution of inflammation This indicates that GPR37 in immune cells mediates the anti-inflammatory effects of NPD1 in the context of inflammatory and neuropathic pain (Bang et al., 2018) (**Figure 3**, **Table 2**). These results imply a central role for GRP37 in the resolution of inflammatory pain.

## Concluding Remarks

In conclusion, the above-mentioned studies demonstrate a crucial role for lipids and the corresponding regulatory proteins in processes that lead to persistent and/or neuropathic pain. As pain states are diverse in their respective pathophysiological mechanisms, it became clear in recent years that the contribution of specific lipid mediators to each pain state is also diverse. For example, the linoleic acid metabolite 12,13-DiHOME but not its sister lipid 9,10-DHOME seems to be critically involved in the development of thermal hyperalgesia in CFA- or zymosan-induced inflammatory pain (Zimmer et al., 2018). However, in paclitaxel-induced neuropathic pain, the parent epoxylipids seem to be present in higher concentrations in nervous tissues and seem to be more active than their diol-metabolites (Sisignano et al., 2016). Also, the cellular origin of these lipids may differ in each pain state. Some lipids may be synthesized and released from immune cells as part of the inflammatory response and others may be synthesized and released from neurons upon oxidative or toxic stress.

In recent years, it became clear that the composition and activity of gut microbes influences signaling and communication between gut and brain. Accumulating evidence suggests that alterations in gut–brain signaling may cause pathophysiological changes in the central nervous system that can contribute to the development of diseases such as depression, anxiety, or persistent pain (Mayer et al., 2014).

The contribution of the gut microbiome to persistent pain was described by characterizing germ-free mice in inflammatory pain models. The germ-free mice showed reduced mechanical hypersensitivity in carrageenan-, LPS- or formalin-induced inflammatory pain. Moreover, in germ-free mice reduced concentrations of TNF-α, IL-1b and CXCL1 were observed in carrageenan-induced inflammatory pain (Amaral et al., 2008). Likewise, administration of *Lactobacillus* strains caused induction of cannabinoid (CB2) and opioid receptors in the intestinal epithelial cells in combination with a reduction of visceral pain in a rat model for irritable bowel syndrome (Rousseaux et al., 2007). Indeed, in the gut brain axis, endocannabinoids and their receptors CB1 and CB2 seem to play a crucial role for bidirectional signaling and crosstalk (Sharkey and Wiley, 2016; Russo et al., 2018). In patients with inflammatory bowel syndrome the concentrations of the endocannabinoid anandamide are significantly reduced in inflamed mucosa (Di Sabatino et al., 2011).

These results show, that the gut microbiome can modulate the activity of sensory neurons by influencing the expression of pain relevant cytokines, chemokines, or G-protein coupled receptors. However, further research is required to clarify the role of other lipids and lipid-related pathways in this context.

Likewise, the contribution of lipid-related proteins to specific pain pathologies seems to differ according to the etiology of the respective pain state and to the cell type in which these proteins are active. For example, activation of the BLT2 receptor in sensory neurons was found to cause TRPV1 desensitization and to ameliorate inflammatory pain (Zinn et al., 2017). In contrast, the S1P1 receptor is mostly expressed in astrocytes not in sensory neurons and seems to contribute to neuropathic pain caused by the cytostatic bortezomib (Stockstill et al., 2018) (see **Figure 2**, **Table 2**).

To identify novel targets for the development of analgesics, it is therefore necessary to determine the contribution of each lipid and its associated signaling pathways to each pain state. In this regard, the use of modern technologies, such as tandem mass spectrometry, is extremely helpful and should be adapted as standard analytical method by future studies.

Despite the complex physiological and pathophysiological properties of signaling lipids in persistent and neuropathic pain states, several potentially druggable targets have been identified in recent years. While some proteins, such as GPR37, have just recently been identified as lipid related. Other proteins, such as sEH, are known to be involved in lipid metabolism for many years and inhibitors of sEH have already been tested in clinical trials (Lazaar et al., 2016) and may be available in the near future.

Moreover, lipid-related proteins have been identified in the pathogenesis of neuropathic pain, that can be targeted with already approved drugs, such as CYP2J2 (by telmisartan) (Sisignano et al., 2016) or S1P1 (by fingolimod) (Stockstill et al., 2018). These drugs may easily be repurposed and tested for the treatment of neuropathic pain in patients.

Collectively these results show that targeting lipids and their related proteins is a promising approach in the development of novel analgesics that has already proceeded into clinical testing. However, several questions concerning the above-mentioned lipids and their specific roles in pain pathology remain unanswered. For example, it needs to be investigated if all the above-mentioned lipid mediators are ligands for specific GPCRs. The high number of orphan GPCRs with unknown function or ligand implies, that some of them may be lipid receptors (Tang et al., 2012). Therefore, more research is required to identify specific lipid GPCRs and their respective ligands in pain pathogenesis and to develop pharmacological strategies for targeting lipid mediators or the related proteins with monoclonal antibodies or small molecules for the development of novel analgesics.

## Author Contributions

TO and MS searched the published work, discussed the content, wrote, edited and revised the article, designed all figures and assembled the information for the table. Both authors read and approved to the final draft of the Review.

## Conflict of Interest

The authors declare that the research was conducted in the absence of any commercial or financial relationships that could be construed as a potential conflict of interest.

## Abbreviations

11H-12,13E-LA, 11-hydroxy-12,13-*tans*-epoxy-(9Z)-octadecenoat; 13-/9-HODE, 13-/9-Hydroxyoctadecadienoic acid; 13H-9,10E-LA, 13-hydroxy-9,10-*trans*-epoxy-(11E)-octadecenoate; 13H-9,10-E-LA, 13-hydroxy-9,10-*trans*-epoxy-octadecenoate; 19,20-DHDP, 19,20-Dihydroxydocosapentaenoic acid; 9K-12,13E-LA, 9-keto-12,13-*trans*-epoxy-10E-octadecenoate; AA, Arachidonic acid; AACOCF_3_, Arachidonyl trifluoromethyl ketone; ATP, Adenosine triphosphate; AUDA, 12-(3-adamantan-1-yl-ureido)-dodecanoic acid; BEL, Bromoenol lactone; BLT, B-leukotriene receptor; CaMKII, calcium/calmodulin-dependent protein kinase II; cAMP, cyclic adenosine monophosphate; CB, Cannabinoid receptor; CCI, Chronic-constriction-injury; CCL, chemokine ligand; CFA, Complete Freund’s adjuvant; cGMP, cyclic guanosine monophosphate; CGRP, Calcitonin gene-related peptide; CIPN, Chemotherapy-induced peripheral neuropathy; CMKLR, Chemerin chemokine like receptor; CNS, central nervous system; COX, cyclooxygenase; cPLA_2_, Ca^2+^-activated phospholipase A_2_; CXCR4, C-X-C chemokine receptor type 4; CYP450, Cytochrome P_450_; DHA, Docosahexaenoic acid; DHET, Dihydroxyeicosatrienoic acid; DiHOME, Dihydroxyoctadecenoic acid; DRG, Dorsal root ganglia; DsiRNA, 27mer dicer-substrate silencer RNA; EET, Epoxyeicosatrienoic acid; EIA, enzyme immunoassay; ELISA, enzyme-linked immunosorbent assay; EpOME, Epoxyoctadecenoic acid; ER, Endoplasmatic reticulum; ERK, Extracellular-signal regulated kinase; ESI-LC/MS, electrospray ionization interface liquid chromatography – mass spectrometry; FABP, Fatty acid-binding protein; FACS, fluorescence-activated cell sorting; FFAR, Free fatty acid receptor; G2A, G2 accumulation GPR; GC/EI/MS, gas chromatography electron ionization mass spectrometry; GIRK, G-protein coupled inwardly rectifying potassium channel; GM-CSF, Granulocyte-macrophage colony-stimulating factor; GPCR, G-protein coupled receptor; HETE, Monohydroxyeicosatetraenoic acid; HPETE, Hydroperoxyeicosatetraenoic acid; IB4, Isolectin B4; iCGRP, immunoreactive CGRP; IHC, immunohistochemistry; IKK, Inhibitor of nuclear factor kappa-B kinase; IL, Interleukin; iPLA_2_, cytosolic calcium independent PLA_2_; IκB, Inhibitor of NF-κB; JNK, c-Jun N terminal kinase; LA, Linoleic acid; LC-MS/MS, liquid chromatography–tandem mass spectrometry; LOX, Lipoxygenase; LPA, Lysophosphatidic acid; LPAR, Lysophosphatidic acid receptor; LPC, Lysophosphatidylcholine; LPS, Lipopolysaccharide; LTB4, Leukotriene B_4_; LTP, long-term potentiation; MAPK, Mitogen-activated protein kinases; MCP, Monocyte chemoattractant protein; MELC, multi-epitope-ligand-cartography; MIA, Mono-sodium iodoacetate; MTT, 3(4,5-dimethylthiazol-2-yl)-2,5-diphenyltetrazolium bromide; NDGA, Nordihydroguaiaretic acid; NF-κB, Nuclear factor kappa-light-chain-enhancer of activated B-cells; NICI/GC/MS, negative ion chemical ionization gas chromatography – mass spectrometry; NK, Neurokinin; NMR, nuclear magnetic resonance; NNT, Number needed to treat; NPD1, Neuroprotection D1; NSAID, Non-steroidal anti-inflammatory drug; OGR1, Ovarian cancer G-protein coupled receptor 1; OIPN, Oxaliplatin-induced peripheral neuropathic pain; PBR, Peripheral benzodiazepine receptor; PG, Prostaglandin; PGE_2_, Prostaglandin E_2_; PGG_2_, Prostaglandin G_2_; PGH, Prostaglandin H; PI3K/Akt, Phosphoinositide 3-kinase; PKA, Protein kinase A; PKC, Protein kinase C; PLA_2_, Phospholipase A_2_; PLAG2A, Phospholipase A2, encoded by the PLA2G2A gene; PNS, Peripheral nervous system; PPAR, Peroxisome proliferator-activated receptor; PTGER/EP, Prostaglandin E receptor; qPCR, quantitative real-time polymerase chain reaction; Rho, Ras homolog GTPase; ROCK, Rho-associated, coiled-coil-containing protein kinase 1; RT-PCR, real-time PCR; S1P, Spingosine-1-phosphate; S1PR, Spingosine-1-phosphate receptor; sEH, Soluble epoxide hydrolase; SNL, Spinal nerve ligation; SPM, Specialized pro-resolving mediators; StARD1, Steroidogenic acute regulatory protein 1; STZ, Streptozotocin; tDPPO, trans-diphenylpropene oxide; TLC, thin layer chromatorgraphy; TDAG8, T-cell death associated gene 8; TG, trigeminal ganglion; TGF, Transforming growth factor; TLR4, Toll-like receptor 4; TNF, Tumor necrosis factor; TP, Thromboxane receptor; TPPU, 1-Trifluoromethoxyphenyl-3-(1-propionylpiperidin-4-yl) urea; TRP, Transient receptor potential; TRPA, Transient receptor potential ankyrin ion channel; TRPC, Transient receptor potential canonical ion channel; TRPM, Transient receptor potential melastatin ion channel; TRPV, Transient receptor potential vanilloid ion channel; TSPO, Translocator protein; UPLC-MS/MS, ultra-performance liquid chromatography–tandem mass spectrometry; VEGF, Vascular endothelial growth factor; vlPAG, ventrolateral periaqueductal grey matter; Wnt, Int-1 and related genes.
